# High-throughput super-resolution analysis of influenza virus pleomorphism reveals insights into viral spatial organization

**DOI:** 10.1371/journal.ppat.1011484

**Published:** 2023-06-30

**Authors:** Andrew McMahon, Rebecca Andrews, Danielle Groves, Sohail V. Ghani, Thorben Cordes, Achillefs N. Kapanidis, Nicole C. Robb

**Affiliations:** 1 Biological Physics, Department of Physics, University of Oxford, Oxford, United Kingdom; 2 Kavli Institute for Nanoscience Discovery, Dorothy Crowfoot Hodgkin Building, University of Oxford, Oxford, United Kingdom; 3 Warwick Medical School, University of Warwick, Coventry, United Kingdom; 4 Physical and Synthetic Biology, Faculty of Biology, Ludwig-Maximilians-Universität München, Großhadernerstr, Planegg-Martinsried, Germany; Emory University School of Medicine, UNITED STATES

## Abstract

Many viruses form highly pleomorphic particles. In influenza, virion structure is of interest not only in the context of virus assembly, but also because pleomorphic variations may correlate with infectivity and pathogenicity. We have used fluorescence super-resolution microscopy combined with a rapid automated analysis pipeline, a method well-suited to the study of large numbers of pleomorphic structures, to image many thousands of individual influenza virions; gaining information on their size, morphology and the distribution of membrane-embedded and internal proteins. We observed broad phenotypic variability in filament size, and Fourier transform analysis of super-resolution images demonstrated no generalized common spatial frequency patterning of HA or NA on the virion surface, suggesting a model of virus particle assembly where the release of progeny filaments from cells occurs in a stochastic way. We also showed that viral RNP complexes are located preferentially within Archetti bodies when these were observed at filament ends, suggesting that these structures may play a role in virus transmission. Our approach therefore offers exciting new insights into influenza virus morphology and represents a powerful technique that is easily extendable to the study of pleomorphism in other pathogenic viruses.

## Author summary

Viruses are significant human pathogens. In influenza, virus structure and morphology has been linked to pathogenicity; however commonly used methods to study virus structure are often low through-put, or lack the resolution required to resolve virus particle features. In addition, influenza filaments are frequently understudied due to their fragile nature and loss of filamentous morphology during viral passage. In order to address this, we have developed a fluorescence super-resolution microscopy and rapid automated analysis pipeline to image many thousands of individual influenza virions at a time, gaining information on their size, morphology and protein distribution. Using this method we were able to show that there is no specific alternation of the surface glycoproteins in filaments, and that neuraminidase-enriched Archetti bodies preferentially house viral ribonucleoprotein complexes. Our findings offer new insights into influenza virus particle assembly and virus transmission, and further, our versatile methods have the potential to contribute towards multiple areas of virus research.

## Introduction

Viral disease results in significant illness and deaths in humans each year, and thus represents a large healthcare and economic burden to countries around the world. The COVID-19 pandemic has resulted in the deaths of millions of people, while annual influenza epidemics can result in up to 650,000 respiratory deaths per year [1], with significantly more during intermittent influenza pandemics. Despite the high mortality and morbidity associated with viruses, many aspects of their structure and morphology are poorly understood, in part due to their pleomorphic nature and small size, which represent a challenge when it comes to imaging and studying them via conventional means.

Influenza virus particles are highly pleomorphic [2], ranging in size from spherical virions ~ 100 nm in diameter [3] to filaments of a similar width but reaching many micrometers in length. Similar pleomorphism has been seen for many other viruses, such as Respiratory Syncytial Virus (RSV) [4], Ebola [5], measles [6], human parainfluenza virus type 2 (HPIV2) [7] and Newcastle Disease Virus [8]. Filamentous strains of influenza have frequently been overlooked in virus research, both because laboratory-passaged viruses tend to be spherical (with a filamentous morphology more typical of clinical isolates) and also because ultracentrifugation and other purification and storage procedures tend to damage filaments [9]. Filamentous virus structure is of interest as it has been linked to increased pathogenicity [10–12], resistance to neutralizing antibodies [13] and penetration through host mucus barriers [14], as well as offering insights into virus assembly and mechanisms of infection [15].

Influenza virus particles are surrounded by a lipid bilayer, in which two surface glycoproteins, the hemagglutinin (HA) and neuraminidase (NA), as well as the M2 protein, are embedded [16]. The M2 protein has ion channel activity, which results in acidification and subsequent uncoating of the viral genome when viruses are internalized into host cells. In addition to ion channel activity, the M2 protein plays a role in virus assembly by binding to M1 [17]. Electron micrographs of spherical viruses have suggested that there are ~375 surface protein spikes, of which approximately one seventh are NA and the rest are HA [16,18]. The distributions of the HA and NA glycoproteins over the virion surface are not entirely random; with studies showing that small clusters of NA are formed within the more abundant HA [16], that NA has a tendency to cluster at one of the poles of filaments [14,16,19–21] and that HA and NA proteins may alternate on the filament surface [14]. Beneath the lipid bilayer lies the structural matrix protein M1—in filaments, this has been observed to have a helical structure [21] and has been implicated as a major determinant of the pleomorphic structure of influenza [22]. Other studies have suggested that the M2 protein may also play a role in filament formation [23,24], and treatment of influenza A virus-infected cells with an M2-specific antibody has been shown to result in loss of filament formation [25].

Inside the M1 core lies the viral genome, which is comprised of 8 viral RNA segments, each of which is bound by multiple nucleoproteins (NP) and the RNA polymerase (formed of the subunits PB1, PB2 and PA) to make ribonucleoprotein (RNP) complexes. Imaging work has suggested that RNP complexes cluster at one end of filamentous virus particles [14,19–21,26]. Some filamentous virions have been observed to have large bulges at one end known as Archetti bodies [27]; previous electron microscopy images have indicated that these bulges do not appear to contain RNPs [20] and their exact purpose in viral replication remains unknown.

The imaging of viruses by conventional fluorescence microscopy is limited as virus particles are generally smaller than the ~ 250 nm resolution limit of optical microscopy for visible light, making it impossible to gain detailed information on the shape, size and protein organisation of individual virions. Electron microscopy (EM), and more recently cryo-EM, are excellent methods for high-resolution imaging of virus structure, but can be comparatively low throughput, time consuming, and offer limited molecular identification. The pleomorphy of many viruses also precludes visualization by procedures that rely on averaging many identical particles, and as a result, it remains challenging to measure protein distributions and abundance at the single-virus level across an entire population. To overcome those limitations and facilitate the high-throughput acquisition of images of filamentous influenza virions, we used direct stochastic optical reconstruction microscopy (dSTORM) [28], a technique that allows the location of molecules to be determined with a resolution of less than 20 nm and offers an exciting alternative for virus imaging [29,30]. This method is compatible with immunostaining, allowing us to specifically label multiple viral proteins within virions.

In this study, we have combined dSTORM imaging methodology with rapid automated analysis software to carry out a high-throughput and high-resolution analysis of thousands of virions at a time. We have used these tools to investigate the structure and organization of influenza H3N2 A/Udorn/72; a strain which exhibits both spherical and filamentous morphology. We found that length analysis provided a useful way of characterizing virions: filaments longer than ~ 230 nm formed a broad size distribution, while smaller particles formed two distinct populations, likely corresponding to spherical and elongated virions. In contrast to previous analyses [26], axial ratio analysis did not reveal distinctive populations. We also investigated the arrangement of viral proteins; demonstrating that no generalized spatial frequency patterning of HA or NA on the virion surface occurs, and observed that RNPs are preferentially located at filament ends when Archetti bodies are present. Our analysis pipeline is versatile and can be adapted for use on multiple other pathogens, as demonstrated by its application to SARS-CoV-2. The ability to gain nanoscale structural information from many thousands of viruses in just a single experiment is valuable for the study of virus assembly mechanisms, host cell interactions and viral immunology, and should be able to contribute to the development of viral vaccines, anti-viral strategies and diagnostics.

## Results

### High-throughput imaging of influenza using super-resolution microscopy

**Fig 1:**
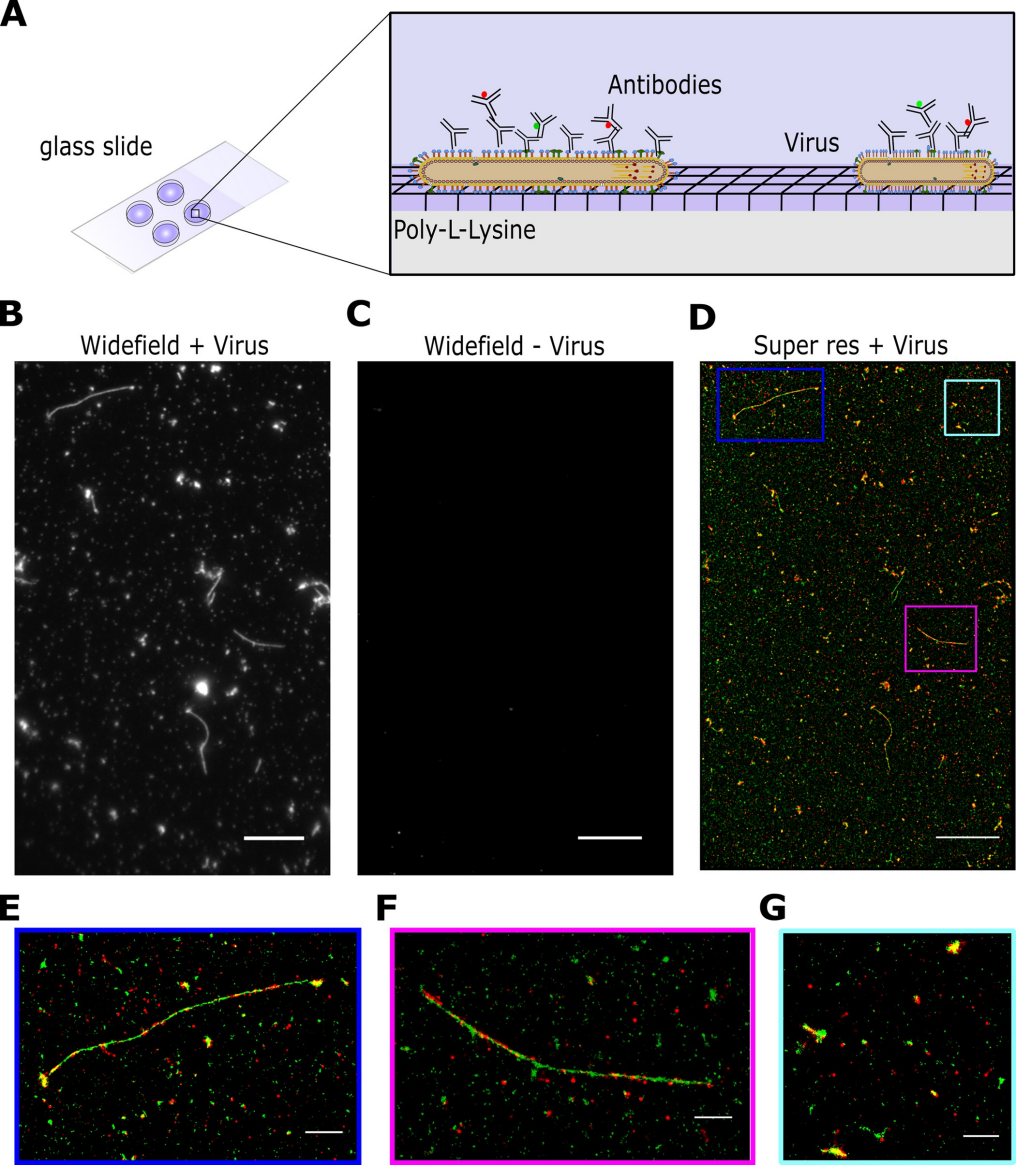
High-throughput imaging of influenza using super-resolution microscopy. A) Schematic of the labelling protocol. Virus samples were dried directly onto glass coverslips pre-coated with poly-L-lysine before being fixed, permeabilised and stained with antibodies using a standard immunofluorescence protocol. B) A representative field of view (FOV) of a widefield image of labelled A/Udorn/72 influenza, imaged in the green channel. Scale bar 10 μm. C) A representative FOV of a widefield image of a virus negative sample, imaged in the green channel. Scale bar 10 μm. D) The corresponding dSTORM image of the FOV in B), where HA is labelled in green and NA is labelled in red. Scale bar 10 μm. E-G) Zoomed in images from D) showing individual filaments and spherical particles. Scale bar 5 μm.

In order to establish a rapid, robust and high-throughput method of imaging virus particles we used influenza A/Udorn/72, an influenza strain with a well-characterized spherical and filamentous phenotype [20]. We initially immobilised virus particles for fluorescence imaging by non-specifically biotinylating the virus surface and incubating particles on the surface of a pegylated glass slide, however this method did not result in many immobilized particles (S1A Fig), was not consistent, and required time-consuming slide preparation. To address these issues, we investigated virus immobilisation via coating the glass with the positively-charged linear polymers poly-L-lysine (S1B Fig) or chitosan (S1C Fig). Both chitosan and poly-L-lysine immobilised virus particles well and with low background, however poly-L-lysine was chosen for subsequent experiments due to its long-term stability and ease of preparation. Virus samples were dried directly onto glass coverslips pre-coated with poly-L-lysine, a process which took approximately 10 minutes (Fig 1A). We found that drying of the virus did not visibly affect filament appearance and that a combination of drying the sample and using poly-L-lysine increased the number of immobilized viruses on the slide surface and the accessibility of long filaments to antibody staining (S2A–S2D Fig). Immobilised viruses were fixed, permeabilised and stained with antibodies using a standard immunofluorescence protocol (see methods). Whilst post-permeabilisation staining and fixation resulted in a slightly higher particle count per field-of-view, we chose to fix first, permeabilise and then immunostain as this allowed us to work with fixed, inactivated viruses from the outset of the experiment and still resulted in a high particle count (S2E Fig).

Next, we imaged the immobilized virus particles using a widefield total internal reflection fluorescence (TIRF) microscope. A/Udorn/72 virions were dual-stained using polyclonal anti-HA and anti-NA primary antibodies and secondary antibodies labelled with Alexa546 (green) and Alexa647 (red) respectively (Fig 1B). A single field-of-view (FOV; measuring 50 x 80 μm) showed examples of multiple viruses, including large numbers of spherical particles and filaments of a wide range of different lengths (Fig 1B). A negative control consisting of media from non-infected cells showed that the labelling was specific (Fig 1C). A long acquisition (10,000 frames) of the FOV was taken to generate a super resolution image, which showed the virus particles at high-resolution, revealing the spatial organization of HA and NA on the surface of both spherical particles and filaments (Fig 1D–1F). Multiple FOVs of the same sample can be imaged in this way, providing high-resolution information on thousands of spherical influenza virions and hundreds of filaments in each run. Taken together, this approach allows us to efficiently, rapidly and easily immobilize and image large numbers of virus particles at high-resolution, in order to gain structural information on large virus populations.

### Length analysis of long filaments using diffraction-limited microscopy images

**Fig 2:**
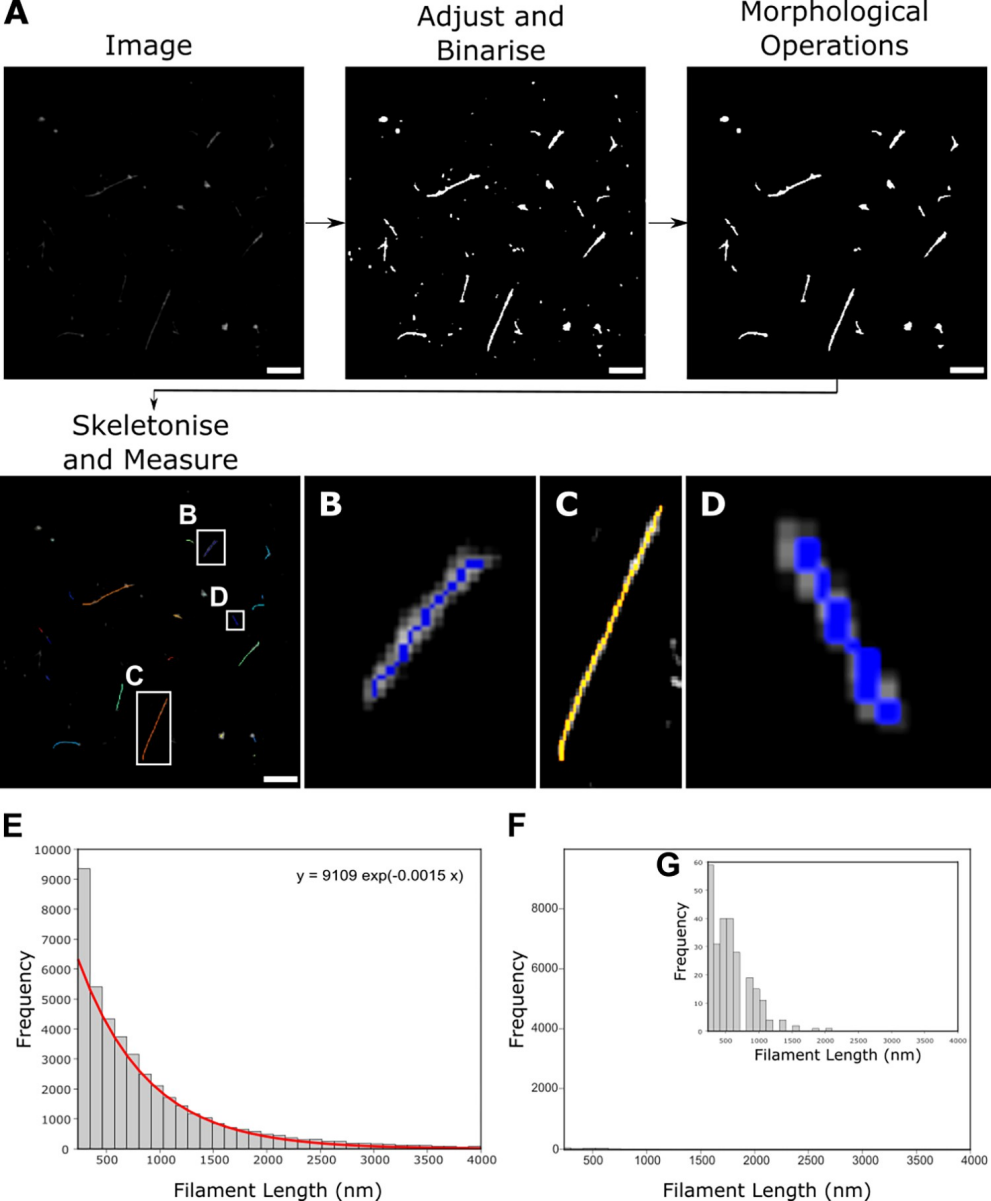
Widefield imaging and automated length analysis of A/Udorn/72 reveals that filaments fit a single distribution. A) A pipeline for analyzing filamentous Udorn particles uses multiple widefield images, which are adjusted and binarised before being skeletonized to get a measure of filament length. Scale bar 5 μm. B, C&D) Zoomed in skeletons from A). E) Widefield images of A/Udorn/72 virus stained with an antibody against the HA protein were analysed using the pipeline in A). The resulting frequency distribution of the lengths of 46,872 filamentous particles, fit with a double exponential (red line; equation above plot). F) Histogram from the analysis of 243 virus-negative FOVs demonstrates that the background was negligible. G) Zoomed in histogram of F).

Our initial images showed a population of elongated virus particles with large variability in length, ranging from approximately 250 nm to several microns. Due to the large size of the viral filaments (larger than the diffraction limit), we initially performed a high-throughput length analysis of multiple filaments using widefield images containing diffraction-limited signals. Multiple images of A/Udorn/72 virus labelled with an anti-HA antibody were taken and a rapid automated analysis pipeline was used to measure filament length (Fig 2A). We adjusted and binarized each image to pick out the virus filaments (Fig 2B, 2C and 2D), using a lower threshold of 234 nm to exclude any signals less than 2 pixels in size. Morphological closing operations were used to fully connect the virus particles if there were regions without labelled protein and then a skeletonization operation [31] which reduced the particles to the simplest shape to give the shape of the filaments was applied. Morphological closing operations have a risk of merging particles but, in general, we observed that filaments were not abundant enough in each FOV to be merged together, the lengths of the resulting skeletons were therefore taken as the lengths of the virus filaments [32,33].

This process was completed on 486 individual FOVs, allowing the lengths of 46,872 filamentous particles to be measured. The resulting histogram was fitted with a single exponential function (Fig 2E). Repeating the process on 243 FOVs negative for virus showed that any background signal due to spurious noise or background labelling was negligible (Fig 2F and 2G) and could be subtracted from the histogram of viral lengths. The resulting length distribution revealed that there were significantly more filaments of shorter lengths (<1000 nm), with the frequency of filaments decreasing at longer length scales. This is consistent with previous observations that filaments are fragile [9] and can break or fracture at longer lengths; alternatively, length may be limited by the greater amount of membrane and viral proteins required to form each filament compared to a spherical particle. The large variability in filament lengths and lack of distinctive sub-populations of filamentous virions of a particular size also suggests a model of virus particle assembly where membrane scission and the release of progeny filaments from cells occurs in a stochastic way, as suggested previously [14].

### Size and shape analysis of virions using dSTORM

**Fig 3:**
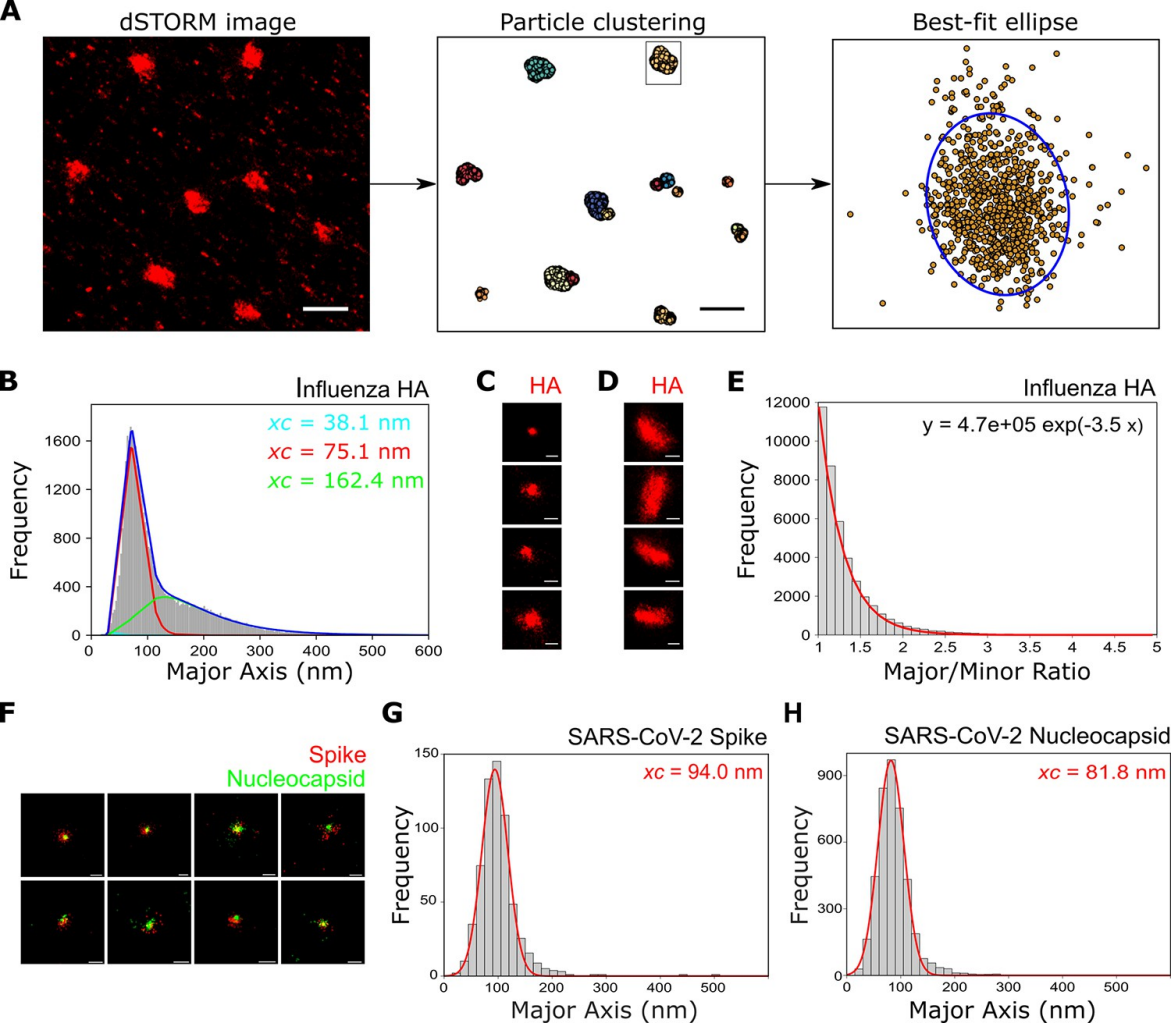
Super-resolution imaging and size analysis of spherical virus particles. A) A pipeline for analyzing spherical and bacciliform particles using multiple super-resolution images. Super-resolution localisations are clustered using DBSCAN and each cluster is fitted with an ellipse. Particle dimensions are extracted using the major and minor diameters of each ellipse. Scale bars 1 μm. B) A histogram of the major axis lengths shows that influenza virions stained for the HA protein fall into two distinct populations, centered at 75.1nm and 162.4 nm. C) Representative particles from the population centered at 75.1 nm. Scale bar 100 nm. D) Representative particles from the population centered at 162.4 nm. Scale bar 100 nm. E) Histogram of the major/minor axis ratio of influenza particles shows a single distribution. F) Representative super-resolution images of SARS-CoV-2 virions dual-labelled with anti-spike and anti-nucleocapsid primary antibodies and secondary antibodies labelled with Alexa647 (red) and Alexa546 (green) respectively. Scale bar 100 nm. G) A histogram of the spike protein major axis lengths fitted with a Gaussian function shows that SARS-CoV-2 virions fall into a single population, centered at 94.0 nm. H) Analysis of the nucleocapsid protein also falls into a single population centered at 81.8 nm.

Having completed a comprehensive length analysis of long virions we next considered how to do this for smaller virus particles (specifically, those that fall into the spherical, ellipsoidal/bacilliform and short filament (<600 nm) categories). The small size of these viruses precluded the use of widefield images to get accurate length data, and so we turned to super-resolution images to obtain their size distribution. A/Udorn/72 virus was immobilized, stained and imaged using dSTORM as described above; the DBSCAN clustering algorithm [34] was used to cluster the resulting localisations in each FOV, and a best-fit ellipse was fitted to each cluster (Fig 3A). The best-fit ellipses were used to give the major and the minor axis lengths of the clusters, which were taken as a measure of virus particle size and the results were plotted as histograms.

This process was completed on 46 individual FOVs, allowing the size of 41,754 particles to be measured. By repeating the process on virus-negative FOVs, the data was corrected for noise or background (S3 Fig). The resulting histogram of the major axis length was fitted using a triple log-normal function, chosen to take into account the two distinctive virion populations as well as a minor third distribution representing the small amount of background signal still remaining (Fig 3B). This analysis revealed two distinct populations of virions (Fig 3C and 3D), centered at ~ 75 nm (54.6% of the total particles) and ~ 160 nm (44.2% of the total particles), likely corresponding to spherical and elongated virions respectively. The third distribution, representing 1.2% of all particles, was centered at ~ 40 nm; the extremely small size of this distribution suggests that our clustering methods and subtraction of data points from virus-negative experiments were sufficient to remove most non-specific localisations. We plotted the precision error in individual localisations for the super-resolution images used in this analysis, which gave a median error of 7.4 nm in the x and 7.2 nm in the y direction, too small to make any meaningful difference to our results (S4 Fig).

EM images of virus particles have previously been categorized based on their axial ratio (<1.2 for spherical virions, >1.2 for bacilliform virions and filaments), as well as length. We therefore plotted the major/minor axis histogram from the measured particles fitted with a single exponential function (Fig 3E), as well as the major axis against minor axis (S5A and S5B Figs). Rather than being able to distinguish two distinct axial ratio populations (of greater than and less than 1.2), we observed a single population. This suggests that when analysing large numbers of virions (many thousands compared to a few hundred via EM) a much larger variability in particle shape may be captured, thus suggesting that a greater heterogeneity in virus shape than previously thought may occur.

In order to demonstrate the versatility of our imaging and analysis pipelines, we carried out similar experiments on the SARS-CoV-2 virus, the causative agent of the COVID-19 pandemic. Fixed SARS-CoV-2 virions were dual-labelled for the spike (red) and nucleocapsid (green) proteins (Fig 3F); multiple co-localised particles were observed, corresponding to double-labelled virions. An averaged image of a virion made from summing localisations from 44,293 dual-coloured virus particles showed that there was a small alignment offset present between the two channels. The distribution of this composite image shows no bias in any direction, but, as viruses could be imaged in all orientations, it does not necessarily reflect the distribution of individual spike proteins (S6 Fig). Super-resolution localisations for each virion were clustered and each cluster fitted with an ellipse to extract particle dimensions. Analysis of the spike protein localisations showed that virions fell into a single population centered at ~ 94 nm (Fig 3G), in keeping with an expected size for SARS-CoV-2 virus particles [35–38], while the major/minor axis histogram showed a tighter distribution of virion axial ratio than that found for influenza, in keeping with observations that SARS-CoV-2 forms largely spherical particles (S5C and S5D Figs). Repeating the analysis for the nucleocapsid protein revealed a distribution centered around ~82 nm, as expected from an internally labelled structure within the virion (Fig 3H), and the distribution (S5E Fig) measured from virions dual-labelled for the envelope (red) and nucleocapsid (green) (S5F Fig) was centered at ~83 nm, which is slightly larger than the nucleocapsid. All together, these results are in keeping with previous observations on the size and distribution of SARS-CoV-2 virus particles and demonstrate the general applicability of our analysis pipeline to multiple other viruses.

### Surface protein organization through Fourier transform analysis of super resolution images

**Fig 4:**
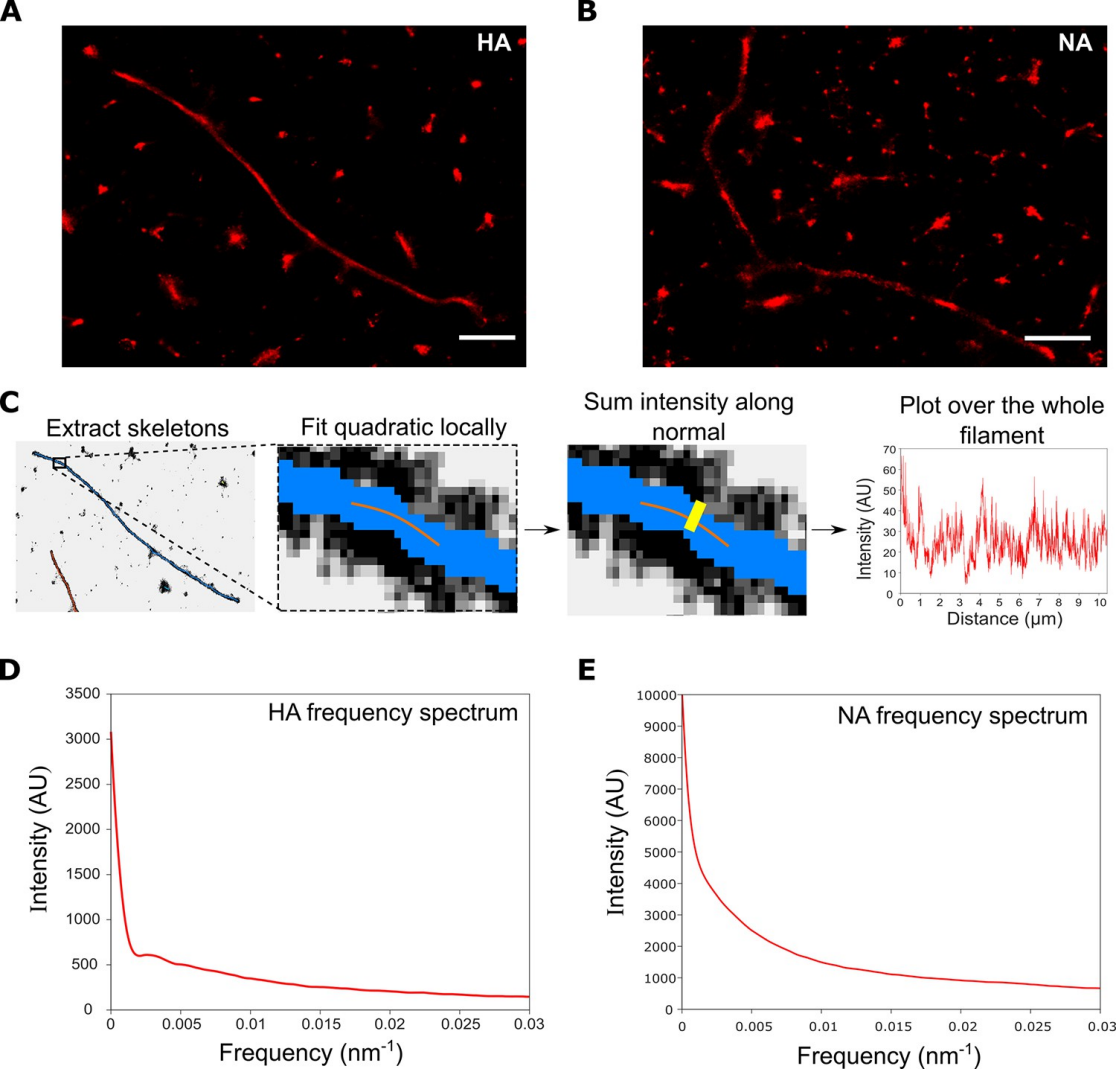
Analysis of the frequency distribution of influenza surface proteins. A&B) Representative super-resolution images of filaments labelled with A) HA antibody and B) NA antibody. Scale bars 1 μm. C) A pipeline for analyzing influenza filament surface protein distribution using multiple super-resolution images. Super-resolution images are skeletonized, fitted with quadratics along the skeletons, the normal is found before the intensity across this normal is summed, and finally a Fourier transform is taken and summed across filaments. D) The frequency distribution of the HA proteins from 1,067 filaments from 8 FOVs. The frequency spectrum for HA has no distinctive peaks, suggesting that there is no common spatial patterning of HA across filaments. E) The frequency spectrum for NA also has no distinctive peaks, suggesting that there is no common spatial patterning of NA across filaments.

The distributions of the HA and NA glycoproteins over the virion surface are not entirely random; indeed, when we stained filaments for either HA (Fig 4A) or NA (Fig 4B) the resulting super-resolution images showed a non-uniform distribution of protein signals. We therefore used intensity analysis and Fourier transforms to investigate the patterning of surface proteins on a large number of filaments. A/Udorn/72 was immobilised, labelled and imaged using dSTORM; the filaments were fit with skeletons as above and the intensity profiles of filaments were found by fitting the skeleton with a polynomial, finding the normal, summing the intensity over the normal and plotting this over the length of the filament (Fig 4C). Finally, the overall patterning was shown by summing the normalised Fourier transforms of all of the filaments, in order to form an average distribution of the patterning.

We started by analyzing the frequency distribution of the HA protein. The resulting distribution showed no peaks apart from the high signal at close to 0 nm^-1^ which is due to the spatial frequency of the filament as a whole (Fig 4D). This suggests that there is no set frequency at which HA proteins are located along influenza virions, which in turn suggests that the surface virion proteins are not patterned deterministically. A modest ‘bump’ at low frequencies (<0.005 nm^-1^) may suggest that individual filaments can contain patterning at a certain spatial frequency, however these frequencies can only be seen in long filaments—the peak suggests patterning on the length scale of >300 nm—and so this is likely to be an artefact of undersampling of longer filaments. Similar analysis was conducted for the NA protein (Fig 4E), which showed no obvious peaks at all, suggesting that no generalized NA spatial patterning on influenza filaments occurs.

**Fig 5:**
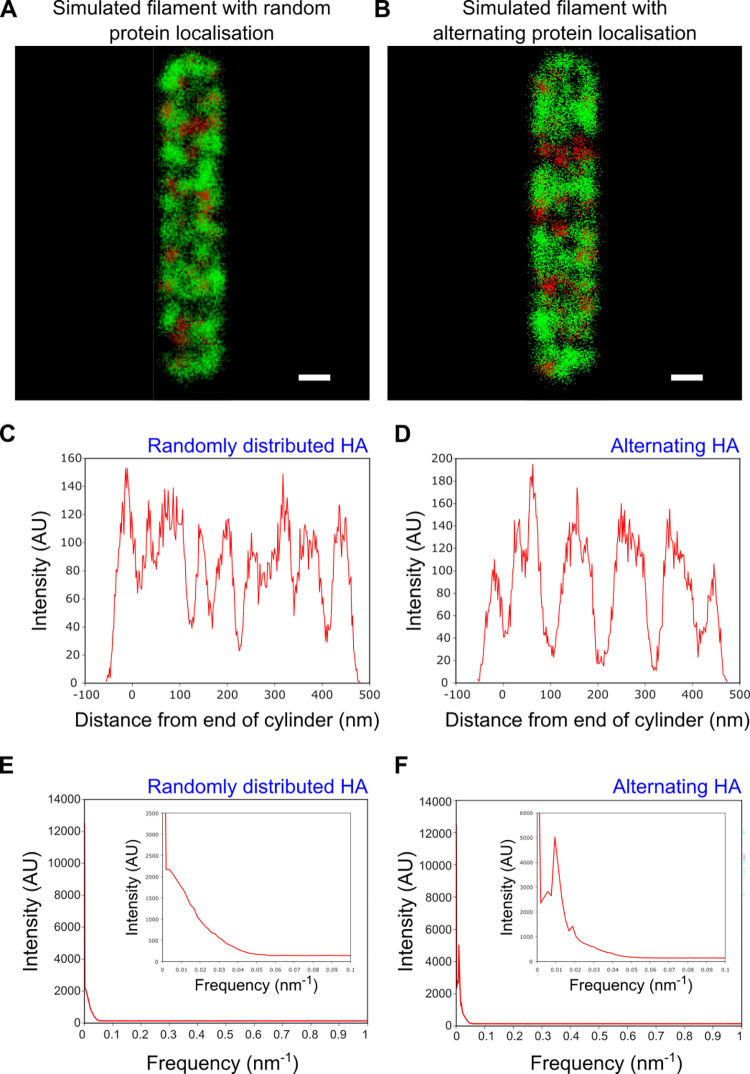
Filamentous virion simulations and analysis of alternation on simulated dSTORM images. A&B) Representative simulated dSTORM images of filamentous influenza particles with A) random protein placement and B) 0.01 nm^-1^ frequency alternating protein placement. HA is shown as green and NA as red, on a filament of 500 nm in length and 80 nm in width. Scale bars 50 nm. C) Intensity profile for a representative simulated filament with randomly distributed HA and NA. D) Same as C) but for filaments with alternating HA/NA. E) The sum of the Fourier transforms of the intensity plots of 1000 simulated filaments with randomly distributed HA and NA, suggesting no common patterning exists. F) Same as E) but for filaments with alternating HA/NA. The peak at 0.01nm^-1^ and harmonic at 0.02nm^-1^ confirm that protein patterning can be detected using this method.

To confirm our observation that no spatial frequency arrangement of the surface proteins occurs, filamentous virions with a length of 500 nm and a diameter of 80 nm were simulated through Monte Carlo methods (S7 Fig). Initially, points corresponding to the HA and NA proteins were positioned randomly on a cylinder with hemispherical caps; by projecting these localisations into two dimensions and randomly fitting points with a normal distribution around the protein positions, simulated dSTORM images could be built up (Fig 5A). For comparison, we simulated dSTORM images of filaments with a forced frequency of protein alternation, representing filaments with a deterministic protein placement (Fig 5B). The relative positioning of the surface proteins in our simulations was confirmed by plotting the HA intensity profiles of representative simulated filaments with either randomly distributed HA and NA, or with alternating HA and NA (Fig 5C and 5D). Next, we investigated the spatial patterning of these simulated filaments by computing the sum of the Fourier transforms of the histograms of protein location along 1000 randomly distributed, and 1000 alternating, simulated filaments. The frequency distribution for filaments with completely randomly chosen points (Fig 5E) was similar to the distributions obtained from experimental data for the HA and NA proteins (Fig 4D and 4E), with no peaks and a broad spread of frequencies at low numbers. The plot for simulations with a forced alternation of 0.01 nm^-1^ however, showed a peak at 0.01 nm^-1^ and a further small peak of the harmonic at 0.02nm^-1^ (Fig 5F). We also showed that the model was capable of detecting alternations smaller than the filament length and also patterning at multiple frequencies (S8 Fig), which again differed from the patterns observed in the experimental data. Together, our data suggests that no deterministic spatial patterning of the HA and NA glycoproteins occurs over a large population of filaments.

### Single-molecule FISH reveals single RNP complexes at the ends of filaments

**Fig 6:**
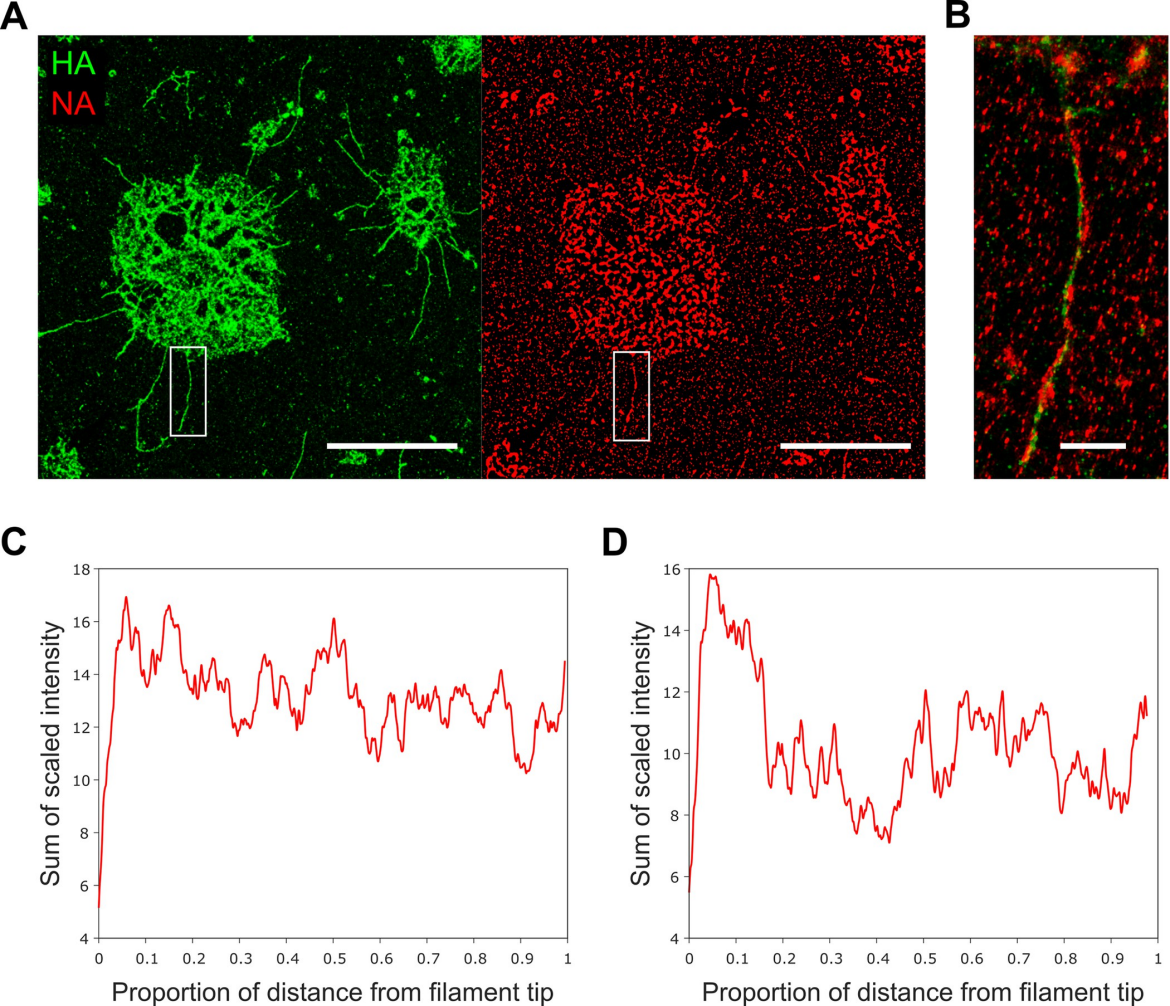
NA is enriched at the tip of budding influenza filaments. A) Diffraction limited images of filamentous A/Udorn/72 stained with for HA (green; left) and NA (red; right) budding out of infected cells. Scale bars 10 μm. B) Zoomed in merged image of filament highlighted in white box in A). Scale bar 1 μm. C) Normalised averaged intensity traces of the HA signal from 34 filaments showing a uniformly distributed distribution along the filament length. D) Normalised averaged intensity traces of the NA signal showing a ~2-fold enrichment at the filament ends furthest from the cell membrane.

Previous work has shown that NA has a tendency to cluster at one of the poles of influenza filaments; however, while tomograms of Udorn virions suggested that NA clusters at the end of the virion opposite to the end where the RNPs are attached [21], a recent analysis using high-throughput fluorescence microscopy to visualize endogenously labelled NA within filaments revealed a tendency for NA to colocalize with NP, in turn suggesting that NA localization is linked to the location of the viral genome [14]. We confirmed previous observations that NA was enriched at one of the filament poles by measuring the intensity profiles of fluorescence signals from immunostained HA (green) and NA (red) in filaments budding out of infected cells (Fig 6A). Budding filaments were clearly defined in the green channel (corresponding to labelled HA), whilst an overlay of the red and green signals showed filaments with largely continuous HA signal punctuated with infrequent clusters of NA (Fig 6B). Using data from both channels as a guide, intensity profiles were measured by drawing a line from the tip of each filament to just before the signal broadened at the cell membrane. The averaged intensity of the HA signal from 34 filaments showed approximately 20% variation in signal intensity, but no polar enrichment (Fig 6C), however, the averaged intensity traces of the NA signal showed a roughly 2-fold enrichment at the filament ends furthest from the cell membrane (Fig 6D). Two of the filaments had large bulges at the tip which we identified as Archetti bodies (S9A Fig). Intensity profiles of these individual filaments showed a strong enrichment of NA signal at the Archetti body (S9B Fig); however, we confirmed that exclusion of these two filaments from our analysis still resulted in stronger NA signal at the filament tip (S9C Fig).

**Fig 7:**
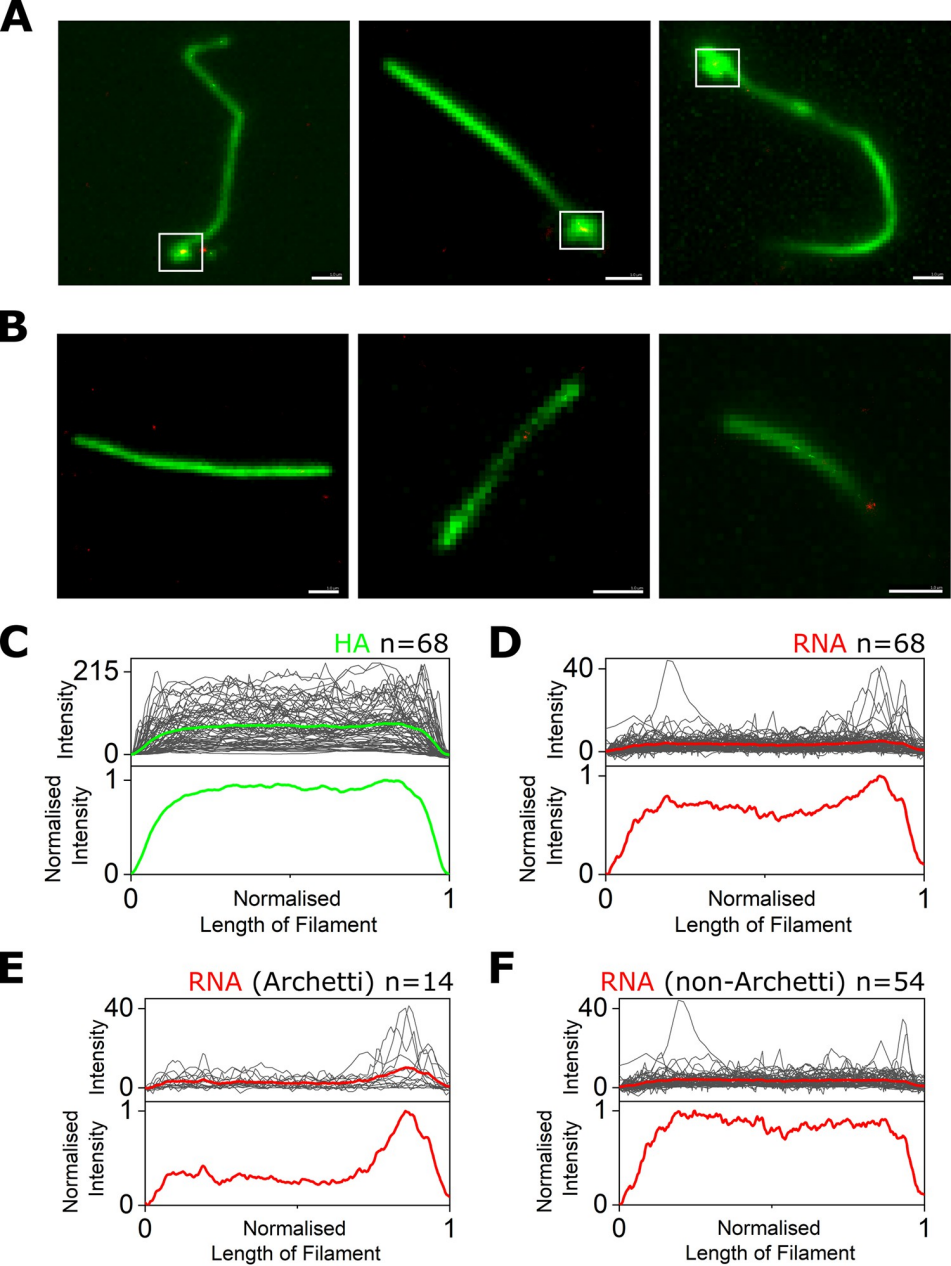
RNA is located at one end of influenza filaments. A&B) Diffraction limited images of filamentous A/Udorn/72 stained with an anti-HA antibody (green) overlaid with super-resolution localisations from an array of FISH probes against the NA gene segment (red). Scale bars 1 μm. White boxes denote Archetti bodies. C) Top: Raw intensity traces (grey) of the HA signal from 68 filaments, with the average intensity profile shown as a green line. Bottom: Normalised average HA intensity from 68 filaments. D) Same as C) but for the red super-resolution signal corresponding to NA RNA from the 68 filaments. E) Top: Raw intensity traces (grey) of the RNA signal from 14 filaments with a visible Archetti body, with the average intensity profile shown as a green line. Bottom: Normalised average RNA intensity from the 14 filaments. F) Same as E) but for the RNA signal from 54 filaments with no visible Archetti body.

Electron microscopy images of bacilliforms and short filaments of influenza have suggested that the RNPs that contain the viral RNA cluster at one end of the virus interior, however clear images of the genome have only been obtained in a minority of longer filaments (reviewed in [9]). In order to clarify the location of RNPs within filaments we used single-molecule fluorescence in situ hybridization (smFISH) to specifically label the influenza NA segment RNA [39] combined with antibody labelling against the HA protein to outline the filament shape. smFISH uses an array of fluorescently labelled DNA probes that bind to several sites on the viral RNA, thereby accumulating several fluorescent dye molecules on a viral RNA, making it visible as a bright spot. A/Udorn/72 virus was immobilized and stained for HA (green) before being incubated overnight with Quasar 670 (red)-labelled FISH probes. Widefield images of the sample showed multiple long filaments of up to several microns in length (green), which were then overlaid with super-resolution localisations from the FISH probes against the NA gene segment (red) (Fig 7A and 7B). Some red localisations not affiliated with virus filaments were observed; we speculate that these are background signal or signal from free RNA not associated with virus particles. 83 filaments were imaged in total, of which 15 were manually discarded as they overlapped with other filaments. Out of the remaining 68 filaments, 14 (20.1%) of these showed a distinctive bulbous shape at one end in the green channel, corresponding to Archetti bodies (Fig 7A; white boxes), while the remaining filaments had no visible bulge at the end (Fig 7B).

In order to determine the position of the RNA in each filament, a line was drawn along the axis of the filaments (using the green HA signal to approximate the filament outline) and the intensity profiles of the line in both the green and red channels was measured. The averaged intensity of the HA signal from all 68 filaments analysed was uniformly distributed over the length of the filaments (Fig 7C), however the averaged intensity traces of the super-resolution signal corresponding to NA RNA from the 68 filaments showed distinct peaks with a tendency to be located towards the filament ends (Fig 7D). This trend was only obvious for filaments that had Archetti bodies (Figs 7E and S11A), compared to those without (Figs 7F and S11B); and all filaments with Archetti bodies had a FISH signal corresponding to viral RNA within the structure, suggesting that Archetti bodies may house RNPs within influenza filaments. The average RNA intensity profile from 23 out of the 54 non-Archetti filaments that had peaks higher than an arbitrary background threshold of 10 did not show any obvious peak at either end of the filament (S11C Fig), and even when the analysis was altered so that all filaments were re-orientated to line up the largest peaks on one side (S11D Fig) there was only a small peak in RNA signal at one end of the filaments. We are therefore unable to conclusively state where RNPs are located within filaments lacking an Archetti body, and speculate that either RNPs don’t have a polarised location in non-Archetti filaments or that some imaged filaments may have lost their Archetti bodies containing RNPs. Together, our results suggest that NA-enriched Archetti bodies may play a role in housing RNPs and potentially in virus transmission.

## Discussion

The small size and pleomorphic nature of viruses precludes their visualization by single-particle averaging methods or conventional fluorescence imaging, making it difficult to measure protein distributions at the single-virus level across an entire population. In this work, we have shown that a combination of super-resolution imaging and high-throughput analysis of virus particles allows for the structural features of multiple virions to be examined at a time. Using the highly pleomorphic influenza strain A/Udorn/72 as a proof of principle, we imaged thousands of filamentous and spherical virions, allowing the features of a large population of virus particles to be ascertained at high-resolution.

Large viral filaments that measured over 250nm in length were analysed using widefield microscopy; analysis of the size distribution of >40,000 filamentous virus in this way showed a broad range of filament sizes. The exact binding epitopes of the antibodies used are unknown, however for HA (the most abundant viral protein on the virus surface and hence the protein most likely to be affected by high density steric hindrance), we observed uninterrupted HA signal along the entire length of the imaged filaments (e.g. Figs 2, 4, and 7), giving us confidence that our results aren’t being affected by epitope accessibility issues. The frequency of filaments decreased with length, suggesting that long filaments are easily broken or that production of longer filaments may be restricted by the need for the large amounts of viral protein required to form each filament compared to a spherical particle. Virion size at short length scales was investigated using super resolution microscopy, where the analysis revealed two distinct size distributions, in keeping with previous observations that influenza forms both spherical and bacilliform particles [8]. The second distinctive population observed is unlikely to correspond to aggregated virus as further populations (corresponding to higher order virus groupings or aggregated virus) in a range of larger sizes were not observed, and we also saw no evidence of virus aggregation in the SARS-CoV-2 experiments, further supporting our conclusion that the two influenza distributions correspond to distinct virion populations. We weren’t able to distinguish between these two populations using their axial ratio, instead observing just a single population of viruses, perhaps because our analysis of such a large number of virions captured a greater heterogeneity in particle shape than previously observed.

Our super resolution method of choice in this study was dSTORM, a single-molecule localization microscopy technique that exploits the ability of photoswitchable fluorophores, attached to antibodies that bind to specific viral protein targets, to stochastically blink. As the number of fluorophores that are in the ‘on’ state is carefully controlled, only a sparse subset is imaged at any one time; hence signals from individual fluorophores can be spatially discriminated. dSTORM achieves super-resolution via the precise localization of the signals emitted from individual molecules by fitting a Gaussian profile, which allows the reconstruction of an image with a localization precision below the diffraction limit. We have identified a number of possible sources of error in this methodology that may contribute to small overestimates or underestimates in our analyses: i) the use of antibodies, which can add an additional linkage error between reporter and epitope which has been estimated to be 10–15 nm [40], ultimately limiting measurement accuracy; ii) the localization error from the Gaussian fit to each localisation during super-resolution image reconstruction, which we calculated to be around 7 nm in our experiments (S4 Fig); and iii) the fitting error from the ellipse fitted to localization clusters to estimate virion size, which may be an underestimate due to the labelling density being greater in the center of a cluster than around the exterior. In a typical dSTORM reconstruction we observed 51±14 HA localisations/nm and 21±13 NA localisations/nm for spherical particles, and 9.8±1.2 HA localisations/nm and 4.1±0.5 NA localisations/nm for filaments (an HA:NA ratio of ~2.4:1 for both spheres and filaments). A previous study suggested an HA:NA ratio of 7:1 per spherical particle [16], suggesting that the labelling efficiency of HA in our experiments could be relatively low, possibly due to steric hindrance of antibody binding. Caution is needed when trying to link dSTORM localisations to protein number however, as the blinking nature of the dyes on the secondary antibodies gives rise to multiple localisations per target, multiple secondary antibodies may bind per target, different antibodies bind to their targets with different efficiencies, and it is unclear whether the surface distribution of glycoproteins and the resulting steric hindrance of antibody binding may differ between spheres and filaments. In spite of these potential sources of error the average sizes that we obtained from our analysis of many thousands of influenza and SARS-CoV-2 particles were in agreement with data obtained through other imaging techniques, confirming that fluorescence super resolution microscopy offers an excellent choice for the rapid and specific study of large numbers of pleomorphic structures without particle averaging.

Our analysis demonstrated large variability in filament length, as well as a lack of distinctly sized sub-populations within a filamentous virus population. In influenza, the initiation of virus budding is thought to be initiated by clustering of HA and NA in lipid raft domains on the cell surface, followed by recruitment of M1 which serves as a docking site for the RNPs just below the plasma membrane [41]. Elongation of the budding virion is caused by polymerization of the M1 protein, and the viral M2 protein is thought to localize at the periphery of the budding virus through interactions with M1 [41]. Finally, membrane scission, mediated by M2, leads to release of the budding virus [42]. Localising M2 within filamentous virions represents an important target for future studies. The regulation of filament formation is not fully understood, but appears to be a complex process driven by at least M1 and M2, and possibly other viral or cellular proteins. Our analysis suggests a model of virus particle assembly where the release of progeny filaments from cells occurs in a stochastic way, leading to filaments of a wide range of lengths. This model is supported by previous observations that influenza produces virions with a broad variation in size and a stochastic protein composition [14], which led to the suggestion that phenotypic variability may contribute to virus survival under stress conditions such as in the presence of antiviral NA inhibitor drugs [43]. Electron microscopy images of influenza virions allow NA and HA spikes on the viral surface to be distinguished by length and density, leading to the observation that HA and NA separate into distinct clusters. Our large-scale analysis showed however that there isn’t a common spatial frequency patterning of HA and NA amongst filaments, suggesting that HA/NA patterning isn’t driven deterministically and further supporting our hypothesis that filament phenotype is broadly variable.

NA has been shown previously to preferentially cluster at the filament pole, either at the same [14] or the opposite [21] end to the RNPs, perhaps to facilitate virus scission and release or directional virus movement. Our analysis confirmed that there was an enrichment of NA signal at the filament tip of budding viral filaments, which has also been suggested to be the location of the RNPs [14,19–21,26]. Interestingly, we also observed a strong NA enrichment in the small number of Archetti bodies found at budding filament tips. In addition, our smFISH analysis showed that RNP complexes were located preferentially at the filament pole within Archetti bodies, in filaments with obvious Archetti bodies at the tip. We believe it is unlikely that the enhanced RNA signals detected in Archetti bodies were due to increased accessibility of FISH probes, as it has been previously show that Archetti bodies have a “contiguous matrix layer” and probes are therefore no more likely to be able to bind at these sites [26]. Together, these results suggest that NA-rich Archetti bodies may play a role in housing RNPs, however, further work is needed elucidate their potential role in virus transmission.

In this work, we have shown that high-resolution microscopy and rapid automated analysis software can be used to gain structural information at the single-virus level on thousands of virions at a time. Although this study introduces these methods on two well-studied viral species with available antibodies against the major viral proteins, there could be general applicability of the techniques to less-well studied virus types, and so our methods may also be useful for future studies of virus assembly and budding, cell-to-cell transmission and virus pathogenesis, or the development of viral diagnostic strategies that rely on virus morphology for identification.

## Methods

### Virus strains

The influenza strain H3N2 A/Udorn/72 (Udorn) was grown in Madin-Darby Canine Kidney (MDCK) cells as described previously [44]. To produce filament-containing stocks for analysis, confluent MDCK cells were infected at a multiplicity of infection of 0.001 and incubated at 37°C in serum-free media (Dulbecco’s Modified Eagle Medium, Gibco) supplemented with 2 μg/mL TPCK-treated trypsin (Sigma) for 24 hours. Supernatants were harvested and clarified at 4000 rpm for 5 minutes at room temperature to remove cell debris before being used without any further purification or concentration steps. 0.2% formaldehyde was added and the supernatant was stored at 4°C to prevent filament degradation. SARS-CoV-2 was grown in Vero E6 cells and collected as above. The virus was inactivated by addition of 4% formaldehyde before use [37] and stored at -80°C.

### Virus immobilization

Unless otherwise specified, viruses were immobilized using poly-L-lysine. Coverslips (25x65mm, thickness number 1, VWR) were heated in a furnace at 500°C for 1 hour to burn off any dust. A silicone gasket (Grace Bio-Labs, USA) was added to the glass slide and 0.01% poly-L-Lysine (Sigma) solution was added to the wells for 30 minutes. The excess was removed and the chambers were washed three times with MilliQ water and the slide allowed to dry. As an alternative chitosan was used in place of poly-L-lysine (0.015% chitosan powder (Sigma) in 0.1M ethanoic acid).

For specific immobilisation of viruses via biotinylation, passivated microscope slides were prepared by washing in acetone and Vectabond solution (Vector Laboratories) before being incubated with NHS-PEG:Biotin-NHS-PEG in an 80:1 ratio. 0.5 mg/mL neutravidin was incubated for 10 minutes at room temperature on the slide shortly before virus was added. Viruses were biotinylated by incubation in a 1 mg/mL Sulfo-NHS-LC-Biotin (ThermoFisher) for 3 hours at 37°C before being fixed and immunolabelled as described below.

### Sample preparation

Virus was incubated in the wells at 45°C until the well was dry. Immobilised virus was fixed with 4% formaldehyde (Thermo Scientific) in phosphate buffered saline (PBS) for 10 minutes at room temperature, before being permeabilised in 0.5% Triton-X-100 (MP Biomedicals) for 15 minutes. The sample was blocked to prevent non-specific binding with 10% donkey serum (Sigma) and 0.1% Tween-20 in PBS for 1 hour at room temperature. Primary antibodies were diluted in blocking buffer and incubated with the sample for 1 hour, followed by washing in PBS and labelling with secondary antibodies (Invitrogen). A final washing step was carried out and samples were stored in PBS at 4°C until imaging. For super resolution imaging, the PBS was replaced with a STORM imaging buffer comprising an enzymatic oxygen scavenging system consisting of 1 mg/mL glucose oxidase and 40 μg/mL catalase, 10% glucose and 0.05 M mercaptoethylamine (MEA) in 1x PBS. Multiple independent repeats, with different virus preparations, were taken over different days.

A/Udorn/72 virions were labelled with the mouse anti-HA primary antibody Hc83x (a kind gift from Stephen Wharton, Francis Crick Institute) and goat anti-NA and anti-M1 (a kind gift from Jeremy Rossman, University of Kent). SARS-CoV-2 virions were labelled with the human anti-spike antibody EY6A [45] (a kind gift from Tiong Tan and Alain Townsend, University of Oxford) and a SARS-CoV-2 nucleocapsid antibody (GTX632269) from Genetex. Secondary antibodies labelled with either Alexa647 or Alexa 546 were purchased from Invitrogen.

For fluorescence in situ hybridisation, the samples were immunostained for HA as described above, then fixed again with 4% formaldehyde in PBS for 10 minutes, washed with 2x saline sodium citrate (SSC) and subsequently permeabilized with 0.5% Triton X-100 in PBS for 10 min. After washing with 2x SSC, the surface was blocked with blocking buffer (2x SSC, 10% formamide, 10% dextran sulfate, 0.02% RNase free-BSA, 0.2 mg/ml E. coli tRNA, 1% RNasin Plus) for 30 minutes at 37°C. The sample was then incubated with 1 μM FISH probes (Stellaris LGC; 48 probes designed against the NA gene segment and labelled with Quasar 670) in blocking buffer for 1-3h at 37°C. After hybridization, the sample was washed thrice with 2x SSC, 10% formamide, 1% RNasin Plus and then washed three times with 2x SSC before being imaged.

### Imaging

All fluorescence imaging experiments were performed on a commercially available Nanoimager fluorescence microscope (Oxford Nanoimaging). The sample was imaged using total internal reflection fluorescence (TIRF) microscopy. The laser illumination was focused at an angle of 53° with respect to the default position. Images of a field of view (FOV) measuring 80 x 50 μm were taken with an exposure time of 30 ms. For super resolution imaging the laser intensities were gradually increased up to a maximum of 780 kW/cm^2^ for both the green (532 nm) and red (647 nm) lasers, and movies of between 5,000 and 15,000 frames were taken. The red channel was imaged first, followed by the green channel.

Drift correction was computed using the inbuilt Nanoimager software, which uses phase correlation between consecutive frames containing a minimum threshold number of localisations to find a translative offset between the two images. In the event that the minimal localization threshold isn’t reached for some later frames in very long acquisitions, there may still be the appearance of minor drift in some clusters in the image. Channel mapping was also computed using the inbuilt Nanoimager software. This uses a mapping file generated from images of a Tetraspek microsphere bead sample which fluoresces in both the red and green channels. The channel offset is found from the phase correlation shift between the two channels and then, using a maximum distance, the software finds colocalised localisations in the channels that do not colocalise with multiple localisations in the other channel. Finally, the mapping matrix is found using least squares minimisation for up to the second order (i.e. 1, x, y, xy, x^2^, y^2^) in the x and y coordinates of the green channel for all colocalised localisations.

### Filament length analysis

Images were saved as.tif files, opened in ImageJ and each FOV was cropped so that just the single channel in which the HA protein was labelled was used. The images were adjusted using the MATLAB imadjust function and binarized using the MATLAB imbinarize function with a threshold of 0.001. The background of the resulting black and white image was removed using the bwpropfilt MATLAB function and the resulting image was closed, skeletonized [31] and labelled using the MATLAB bwmorph, bwskel and bwlabel functions respectively. Any skeletons of less than 234nm (2 pixels) long were excluded from the analysis, as were overlapping skeletons (due to overlapping filaments dried onto the slide). Results were plotted as a histogram and fitted with a bi-exponential function, detailed on the graph.

### Spherical size analysis

Each FOV was drift corrected, and super-resolution localisations were extracted in each frame using the inbuilt Nanoimager software. The localisations were exported and analysed further in Matlab. Localisations were clustered using the sklearn library implementation of the DBScan clustering algorithm with a minimum cluster size of 200 and an epsilon of 30nm [34], thereby excluding large or odd shaped particles. Very small clusters resulting from sub-division of larger particles during the clustering analysis (potentially due to uneven labelling) were filtered out by background subtraction. The clusters were fit using the confidence_ellipse library method of fitting a confidence ellipse to a set of points with 2.0 as the standard deviation of the ellipse. This algorithm uses the covariance of a set of points, the Pearson coefficient and the mean of the points to fit an ellipse to a set of points. The major and minor axes of the ellipse were taken as the size of the viral particles.

### Averaged STORM images

Colocalised clusters between channels were found by clustering and fitting best fit ellipses to the data. The position of localisations in these clusters were taken with reference to either the centroid of one cluster or the centroid of the cluster that a localisation belonged to. These were plotted on heat maps with a pixel size of 3 nm and overlaid in ImageJ.

### Protein distribution and spatial frequency analysis

FOVs with multiple filaments (> 615 nm) labelled with the protein of interest (HA or NA) were skeletonized as in the filament length analysis. For each pixel in the skeleton of filaments, a fit line was produced (using the MATLAB polyfit function) using the next 5 points in each direction from the pixel of interest. From this quadratic, the normal was found and the intensity of the pixels in the normal were summed. After iterating this over the skeleton, the calculated line profile of each filament were Fourier transformed using the MATLAB fft function and the resulting distributions across all filaments were summed to produce the average frequency spectrum for that surface protein [46].

### Virion simulation

dSTORM images of viruses were simulated using a Monte Carlo method with the probability of a protein being placed equally across the virion and an exclusion distance of 20nm. Filaments were modelled as cylinders with hemispherical caps. After projecting the simulated filaments into 2D, localisations were randomly placed with a normal distribution about the protein location with a standard deviation of 7.4nm (derived from the localization precision of our data) and the image was coloured as a STORM image [47]. Alternating proteins were placed by choosing the random number for the protein location along the filament body from probability distributions derived from the functions |sin(kx/2)| and |cos(kx/2)|.

### NA intensity analysis

Supernatant containing both floating cells and virus was harvested from MDBK cells infected with A/Udorn/72 virus was spun onto 8-well plates (Thermo Scientific) at 5000 RPM for 5 minutes to immobilise the sample onto glass. Samples were stained for HA and NA and imaged as described above. Using data from both channels as a guide, a line was drawn in FIJI from the tips of filaments to just before the filament broadened at the cell membrane, with a width slightly greater than the filament width. The lengths of the filaments and intensity profile were normalised (0–1) per filament and summed.

### FISH image analysis

The inbuilt Nanoimager software was used to create dual-colour images of diffraction-limited green fluorescence and super-resolution localisations of red fluorescence of the filaments. To create the intensity along the filament figures, movies recorded at 30 ms exposure with 1000 frames were projected in FIJI to make a single summation image. In FIJI, a line was taken along the axis of the filaments measuring the intensity in the green and red emission channels of the microscope. Filaments containing Archetti bodies were orientated with the Archetti bodies at one end, while filaments without were measured from left to right on the screen, assuming they randomly orientate themselves on the surface. The background intensity (the first value in the intensity line) was subtracted to produce the raw intensity of the fluorescence, the length in pixels was normalised (0–1), and graphs plotted for all lines.

## Supporting information

S1 Fig Immobilisation methods for filamentous viruses. A) A virus negative control (top) or an A/Udorn/72 virus sample (bottom), were immobilized via a specific biotin/PEG linkage. Virus particles were biotinylated by incubation with 1 mg/mL Sulfo-NHS-LC-Biotin (ThermoFisher) for 3 hours at 37°C before being immobilised on a pegylated slide. The virus was labelled with an anti-Udorn primary antibody and an Alexa647 secondary antibody and imaged on a widefield TIRF microscope. Scale bars 10 μm. B) A virus sample was incubated on a slide pre-treated with 0.01% poly-L-lysine. C) A virus sample was incubated on a slide pre-treated with 0.015 mg/mL chitosan in 0.1 M acetic acid.
(JPG)

S2 Fig Optimization of immobilization and sample preparation for imaging viruses. A) A virus negative control (top) or an A/Udorn/72 virus sample (bottom), were incubated on a slide pre-treated with 0.01% poly-L-lysine at 4°C for 10 minutes. The excess sample was removed from the well and the immobilized virus was fixed and labelled with an anti-Udorn primary antibody and an Alexa647 secondary antibody before being imaged on a widefield TIRF microscope. Scale bars 10 μm. B) As in A) but the slide was not pre-treated with poly-L-lysine and the samples were dried directly onto glass coverslips by heating at 45°C for 10 minutes. C) As in A) but the samples were dried directly onto glass coverslips by heating at 45°C for 10 minutes. D) dSTORM reconstructions of Udorn-stained influenza filaments either dried directly onto glass coverslips by heating at 45°C for 10 minutes or incubated on the coverslips at room temperature for 10 minutes. Scale bars 0.5 μm. E) Plot of number of particles detected per diffraction-limited field-of-view (FOV) under different fixation and permeabilization conditions.
(JPG)

S3 Fig Virus-negative FOV and histograms for the size analysis of spherical and bacilliform influenza particles. A) A representative super-resolution image of a virus-negative sample virus stained with an antibody against the HA protein. Scale bar 10 μm. B) Super-resolution localisations were clustered and each cluster fitted with an ellipse to extract particle dimensions. A histogram of the major axis lengths shows that background signal is negligible. C) Zoomed in histogram of B).
(PNG)

S4 Fig Histograms of the localisation precisions of the data from Fig 3. A) Each FOV was drift corrected, and a Gaussian function was fitted to each detected localization in every frame of the acquisition, using the inbuilt Nanoimager software. The fitting error (or localization precision) in the x direction of each localization was exported and plotted as a histogram, providing a median error of 7.4 nm. B) Plot of the localisation precision in the y direction, providing a median error of 7.2 nm.
(JPG)

S5 Fig Super-resolution imaging and size analysis of influenza and SARS-CoV-2 virus particles. A) A plot of the major axis against minor axis for the influenza HA protein. B) A heatmap of the major axis against minor axis for the influenza HA protein. C) A plot of the major axis against minor axis for the SARS-CoV-2 spike protein. D) Histogram of the major/minor axis ratio of spike localisations shows a single distribution. E) Analysis of the envelope protein also falls into a single population centered at 83.0 nm. F) Representative super-resolution images of SARS-CoV-2 virions dual-labelled with anti-envelope and anti-nucleocapsid primary antibodies. Scale bar 100 nm.
(JPG)

S6 Fig The averaged dSTORM structure of the SARS-CoV-2 virion. A) The average structure when centering spike protein localisations on the centroid of nucleocapsid protein. B) The average structure when centering all proteins on their own centroid and aligning these centres. Scale bars 30 nm
(JPG)

S7 Fig Summary of the dSTORM simulation method. Input parameters of virion size, number of localisations and localization precision were used for Monte Carlo simulations to create simulated dSTORM images of filamentous virions. Filaments were modelled as cylinders with hemispherical caps. After projecting the simulated filaments into 2D, localisations were randomly placed with a normal distribution about the protein location with a standard deviation of 7.4nm and the image was coloured as a STORM image. Scale bar 20 nm.
(JPG)

S8 Fig Protein patterning analysis can pick up alternations smaller than the filament length and also patterning at multiple frequencies. A) Frequency distribution from 500 nm simulated filaments with protein frequency patterning at 0.024 nm^-1^; the limit of visible frequencies occurs when the length scales are on the order of half of filament lengths. B) The frequency distribution from 1000 nm simulated filaments with protein frequency patterned at 0.007 nm^-1^ and 0.033 nm^-1^ show patterning at multiple frequencies can be seen. A small peak from a harmonic of the 0.007 nm^-1^ frequency is also observed.
(JPG)

S9 Fig NA is enriched in Archetti bodies as well as at the tip of non-Archetti budding influenza filaments. A) Diffraction limited image of A/Udorn/72 filaments stained with for HA (green) and NA (red) budding out of an infected cell. White line denotes a filament with an Archetti body (arrow) at the tip. Scale bar 1 μm. B) Intensity trace of the NA signal from the filament highlighted in A), showing higher NA signal at the Archetti body at the tip. C) Normalised averaged intensity traces of the NA signal from filaments without obvious Archetti body structures at the tip, showing an enrichment at the filament ends furthest from the cell membrane.
(JPG)

S10 Fig Diffraction limited images of filamentous A/Udorn/72 stained with an anti-HA antibody (green; left panels), super-resolution localisations from an array of FISH probes against the NA gene segment (red; middle panels), and merged images (right panels). White boxes denote Archetti bodies. Scale bars 1 μm.
(JPG)

S11 Fig HA and RNA intensity traces along filaments. A) Top: Raw intensity traces (grey) of the HA signal from 14 filaments with a visible Archetti body, with the average intensity profile shown as a green line. Bottom: Normalised average RNA signal from the 14 filaments, showing peak in intensity of the HA signal in Archetti bodies. B) Same as A) but for the HA signal from 54 filaments with no visible Archetti body. C) Top: Raw intensity traces (grey) of the RNA signal from 23 filaments with intensity peaks above an arbitrary background threshold of 10, with the average intensity profile shown as a red line. Bottom: Normalised average RNA intensity from the 23 filaments. D) Same as C) but for the RNA signal from all non-Archetti filaments that have been re-orientated to line up the largest peaks on one side.
(JPG)
