# Supplementary figures and images for: High-throughput super-resolution analysis of influenza virus pleomorphism reveals insights into viral spatial organization
Source: PLoS Pathog. 2023 Jun 30;19(6):e1011484. doi: 10.1371/journal.ppat.1011484 (PMC10343030; doi:10.1371/journal.ppat.1011484)

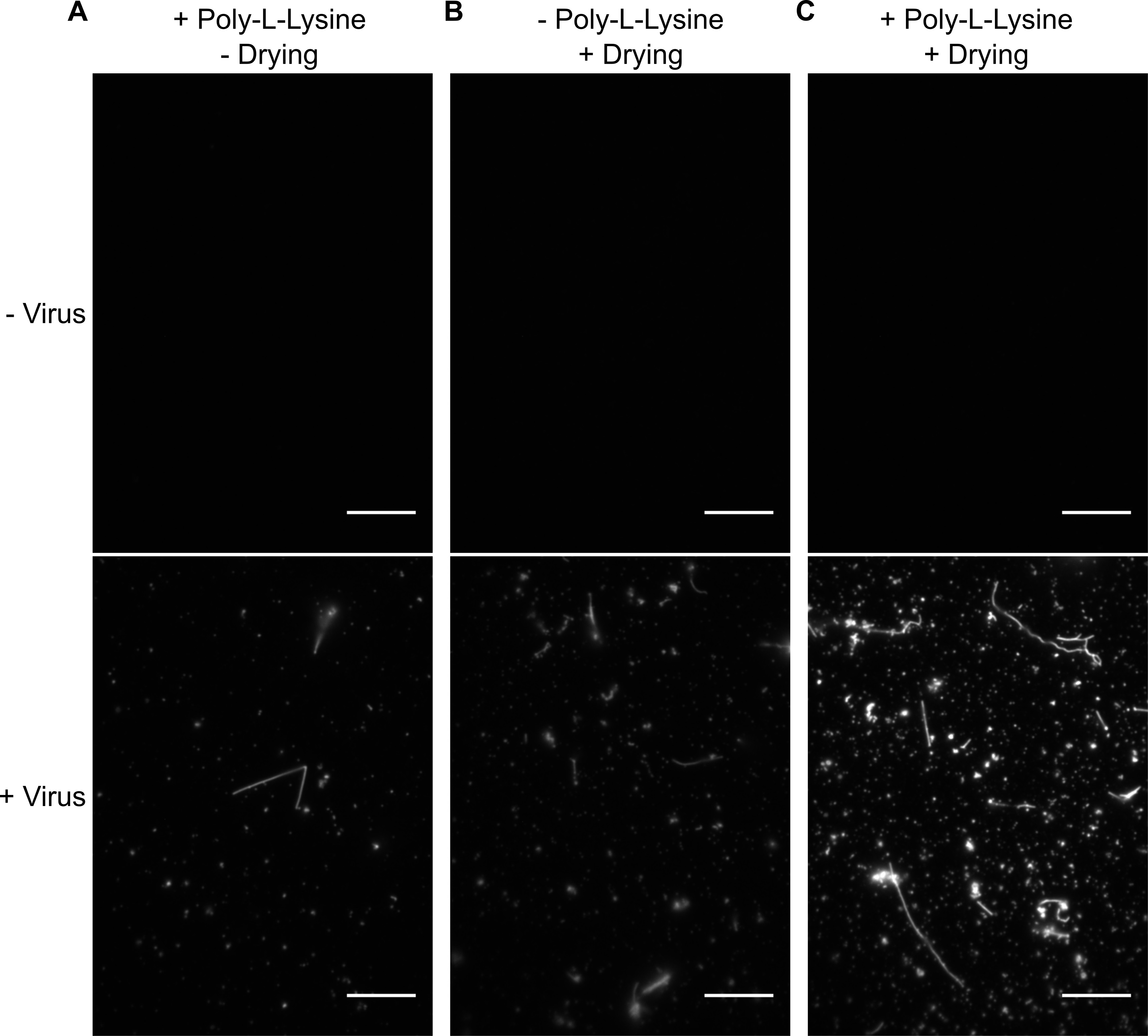

Supplement: S1 Fig — A) A virus negative control (top) or an A/Udorn/72 virus sample (bottom), were immobilized via a specific biotin/PEG linkage. Virus particles were biotinylated by incubation with 1 mg/mL Sulfo-NHS-LC-Biotin (ThermoFisher) for 3 hours at 37°C before being immobilised on a pegylated slide. The virus was labelled with an anti-Udorn primary antibody and an Alexa647 secondary antibody and imaged on a widefield TIRF microscope. Scale bars 10 μm. B) A virus sample was incubated on a slide pre-treated with 0.01% poly-L-lysine. C) A virus sample was incubated on a slide pre-treated with 0.015 mg/mL chitosan in 0.1 M acetic acid. (JPG) [file ppat.1011484.s001.jpg]

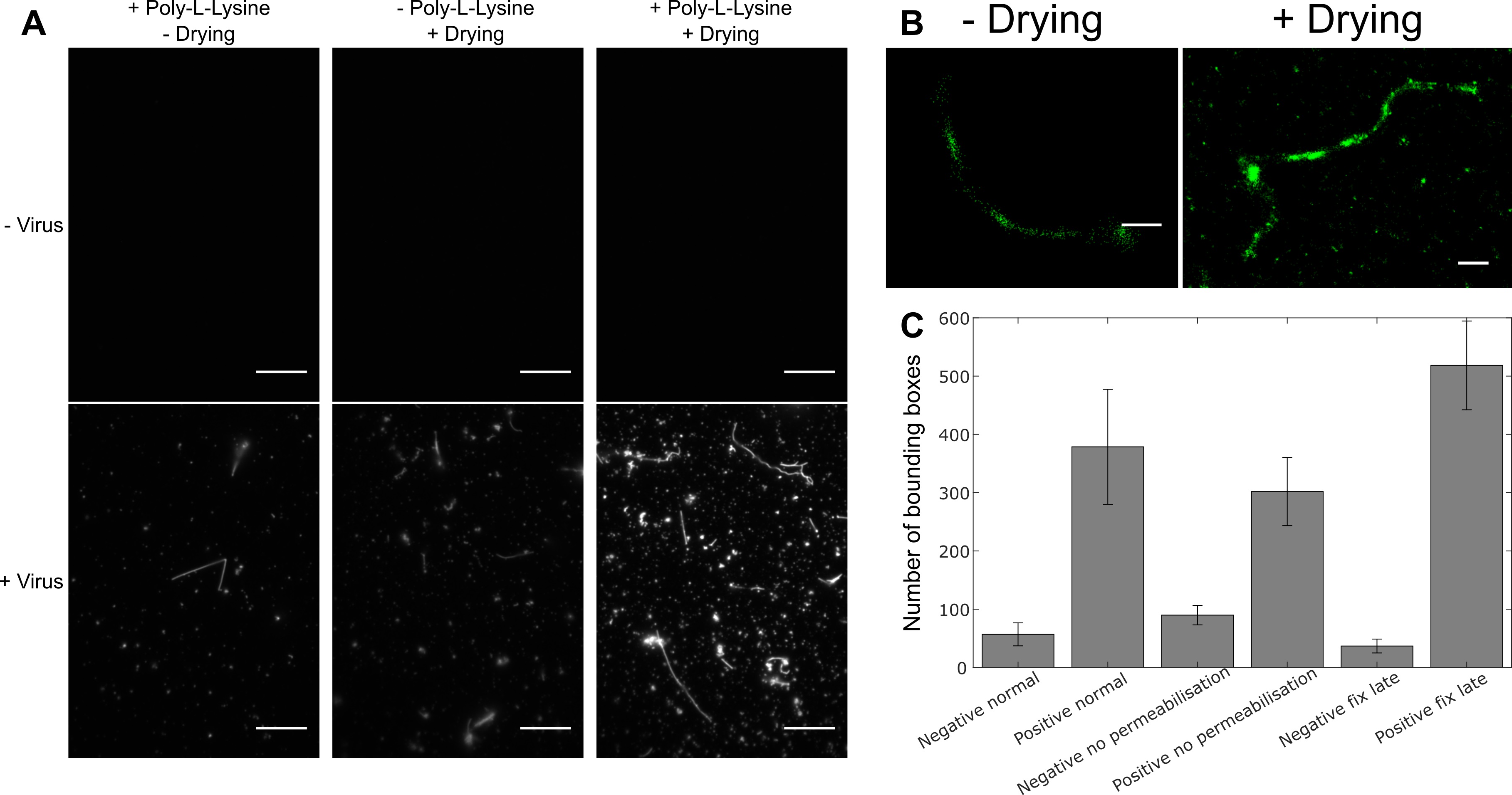

Supplement: S2 Fig — A) A virus negative control (top) or an A/Udorn/72 virus sample (bottom), were incubated on a slide pre-treated with 0.01% poly-L-lysine at 4°C for 10 minutes. The excess sample was removed from the well and the immobilized virus was fixed and labelled with an anti-Udorn primary antibody and an Alexa647 secondary antibody before being imaged on a widefield TIRF microscope. Scale bars 10 μm. B) As in A) but the slide was not pre-treated with poly-L-lysine and the samples were dried directly onto glass coverslips by heating at 45°C for 10 minutes. C) As in A) but the samples were dried directly onto glass coverslips by heating at 45°C for 10 minutes. D) dSTORM reconstructions of Udorn-stained influenza filaments either dried directly onto glass coverslips by heating at 45°C for 10 minutes or incubated on the coverslips at room temperature for 10 minutes. Scale bars 0.5 μm. E) Plot of number of particles detected per diffraction-limited field-of-view (FOV) under different fixation and permeabilization conditions. (JPG) [file ppat.1011484.s002.jpg]

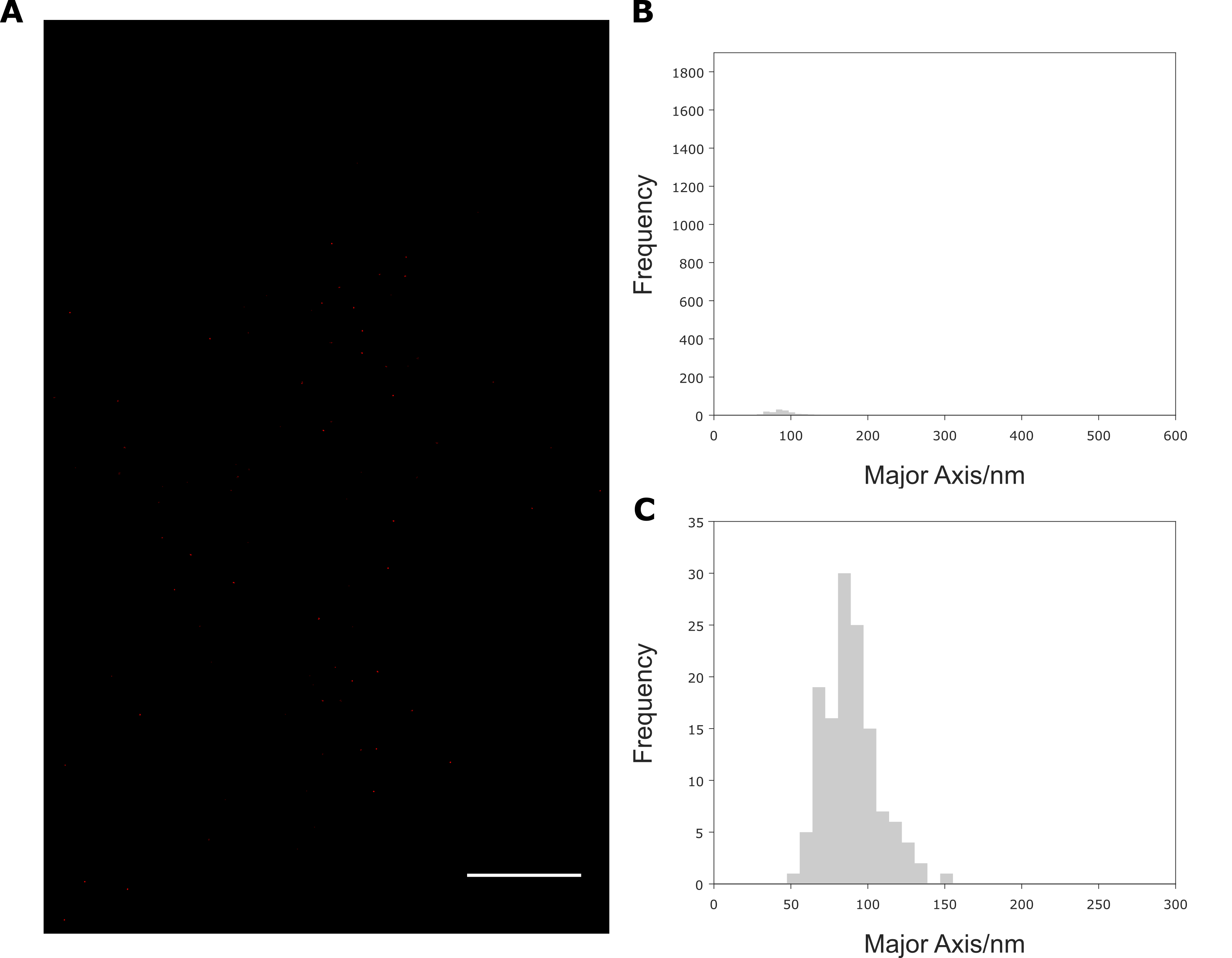

Supplement: S3 Fig — A) A representative super-resolution image of a virus-negative sample virus stained with an antibody against the HA protein. Scale bar 10 μm. B) Super-resolution localisations were clustered and each cluster fitted with an ellipse to extract particle dimensions. A histogram of the major axis lengths shows that background signal is negligible. C) Zoomed in histogram of B). (PNG) [file ppat.1011484.s003.png]

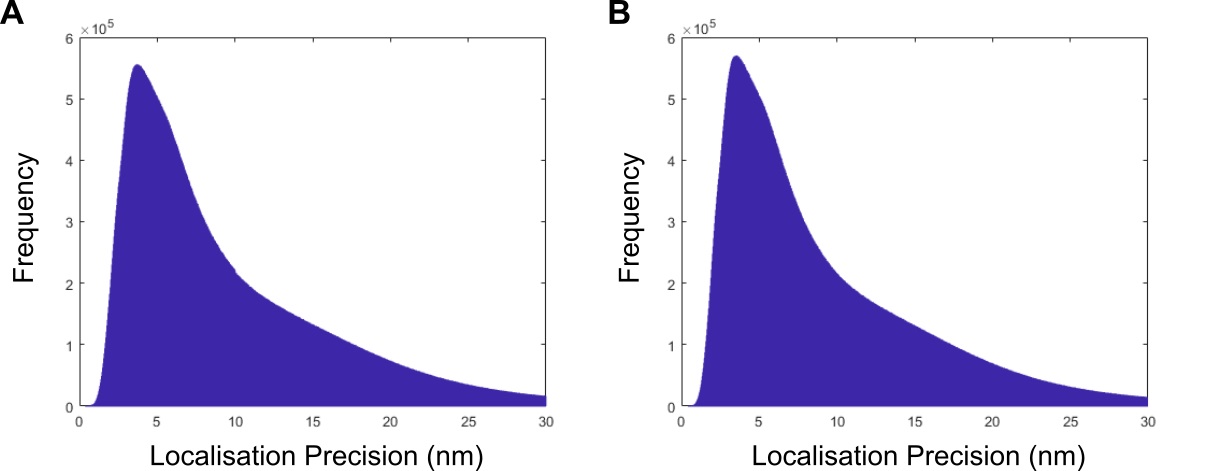

Supplement: S4 Fig — A) Each FOV was drift corrected, and a Gaussian function was fitted to each detected localization in every frame of the acquisition, using the inbuilt Nanoimager software. The fitting error (or localization precision) in the x direction of each localization was exported and plotted as a histogram, providing a median error of 7.4 nm. B) Plot of the localisation precision in the y direction, providing a median error of 7.2 nm. (JPG) [file ppat.1011484.s004.jpg]

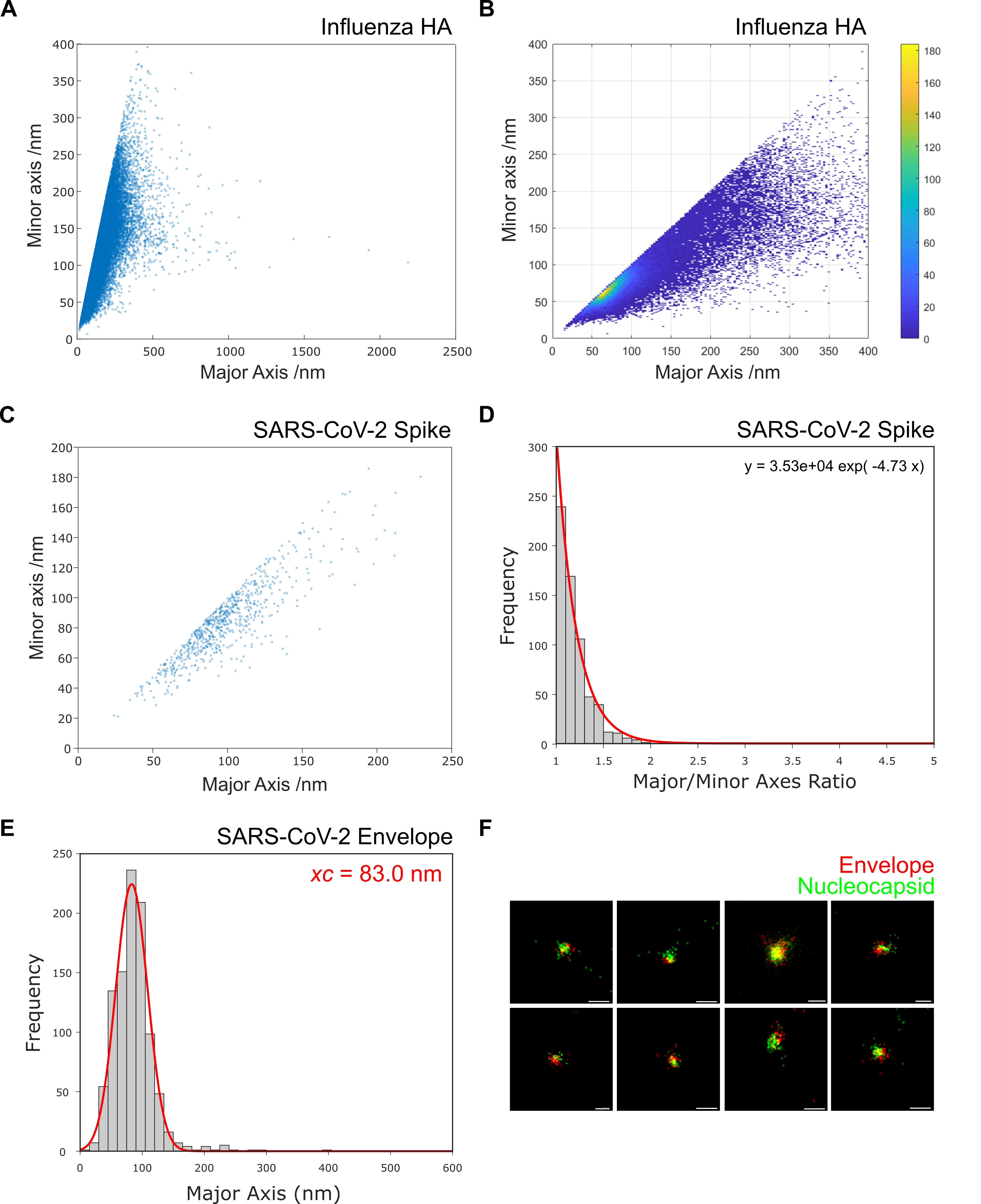

Supplement: S5 Fig — A) A plot of the major axis against minor axis for the influenza HA protein. B) A heatmap of the major axis against minor axis for the influenza HA protein. C) A plot of the major axis against minor axis for the SARS-CoV-2 spike protein. D) Histogram of the major/minor axis ratio of spike localisations shows a single distribution. E) Analysis of the envelope protein also falls into a single population centered at 83.0 nm. F) Representative super-resolution images of SARS-CoV-2 virions dual-labelled with anti-envelope and anti-nucleocapsid primary antibodies. Scale bar 100 nm. (JPG) [file ppat.1011484.s005.jpg]

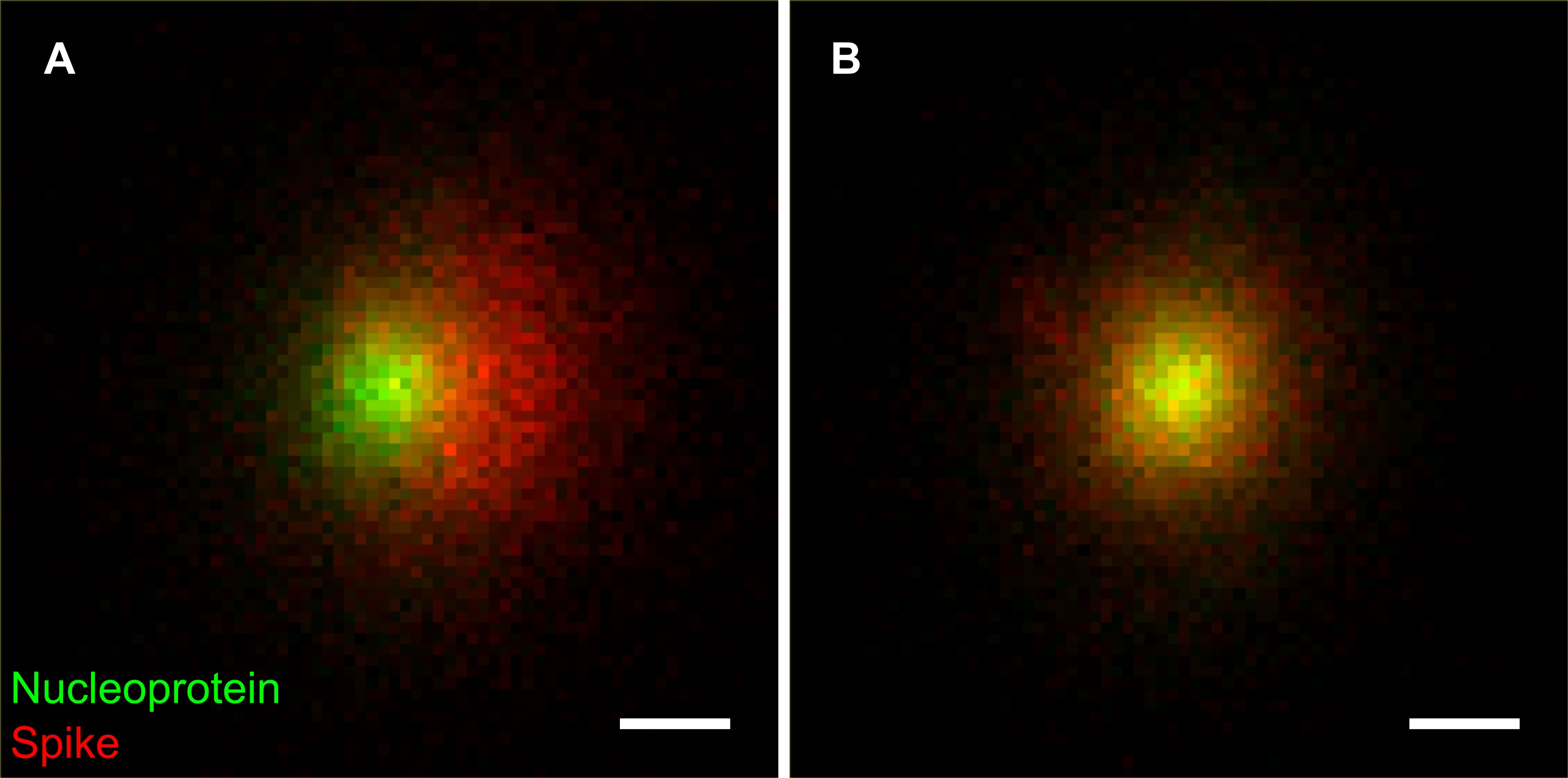

Supplement: S6 Fig — A) The average structure when centering spike protein localisations on the centroid of nucleocapsid protein. B) The average structure when centering all proteins on their own centroid and aligning these centres. Scale bars 30 nm (JPG) [file ppat.1011484.s006.jpg]

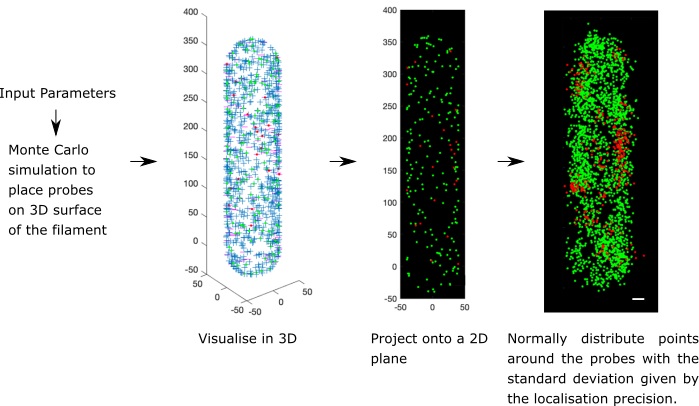

Supplement: S7 Fig — Input parameters of virion size, number of localisations and localization precision were used for Monte Carlo simulations to create simulated dSTORM images of filamentous virions. Filaments were modelled as cylinders with hemispherical caps. After projecting the simulated filaments into 2D, localisations were randomly placed with a normal distribution about the protein location with a standard deviation of 7.4nm and the image was coloured as a STORM image. Scale bar 20 nm. (JPG) [file ppat.1011484.s007.jpg]

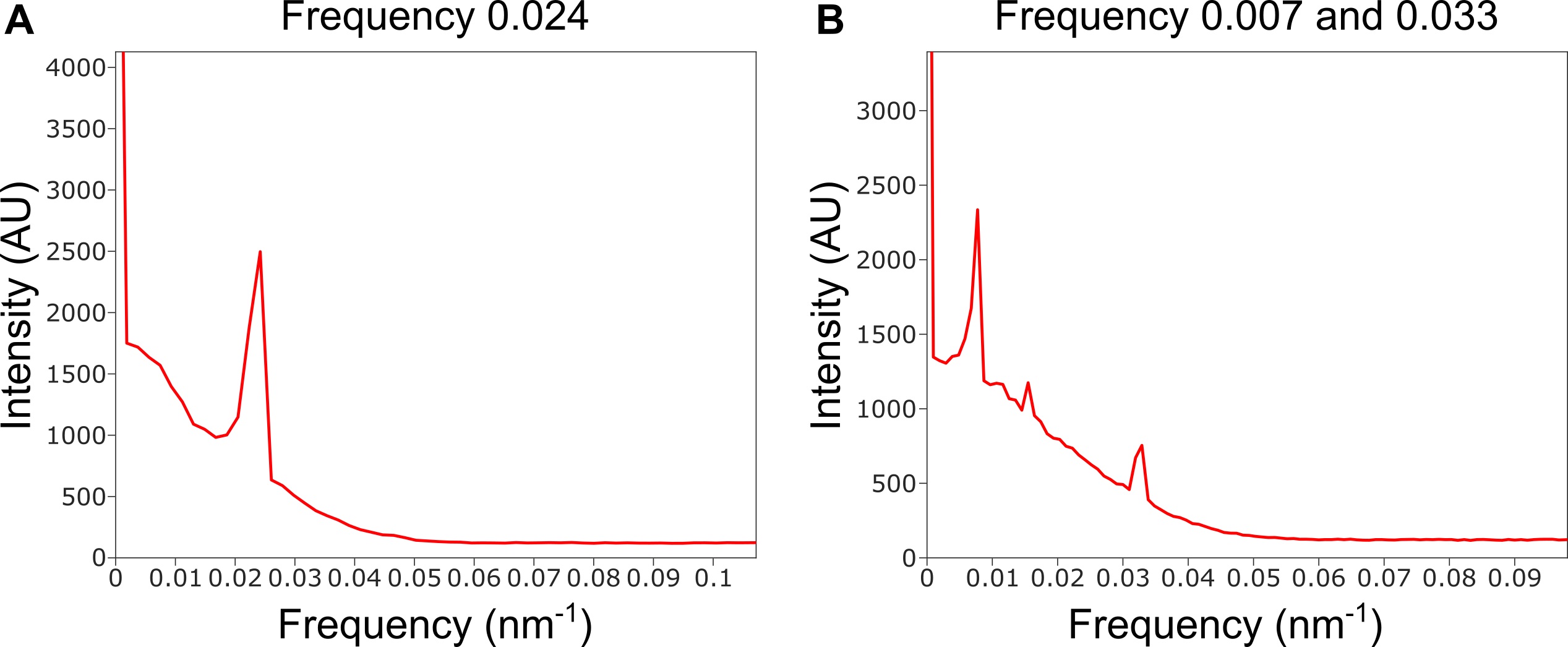

Supplement: S8 Fig — A) Frequency distribution from 500 nm simulated filaments with protein frequency patterning at 0.024 nm-1; the limit of visible frequencies occurs when the length scales are on the order of half of filament lengths. B) The frequency distribution from 1000 nm simulated filaments with protein frequency patterned at 0.007 nm-1 and 0.033 nm-1 show patterning at multiple frequencies can be seen. A small peak from a harmonic of the 0.007 nm-1 frequency is also observed. (JPG) [file ppat.1011484.s008.jpg]

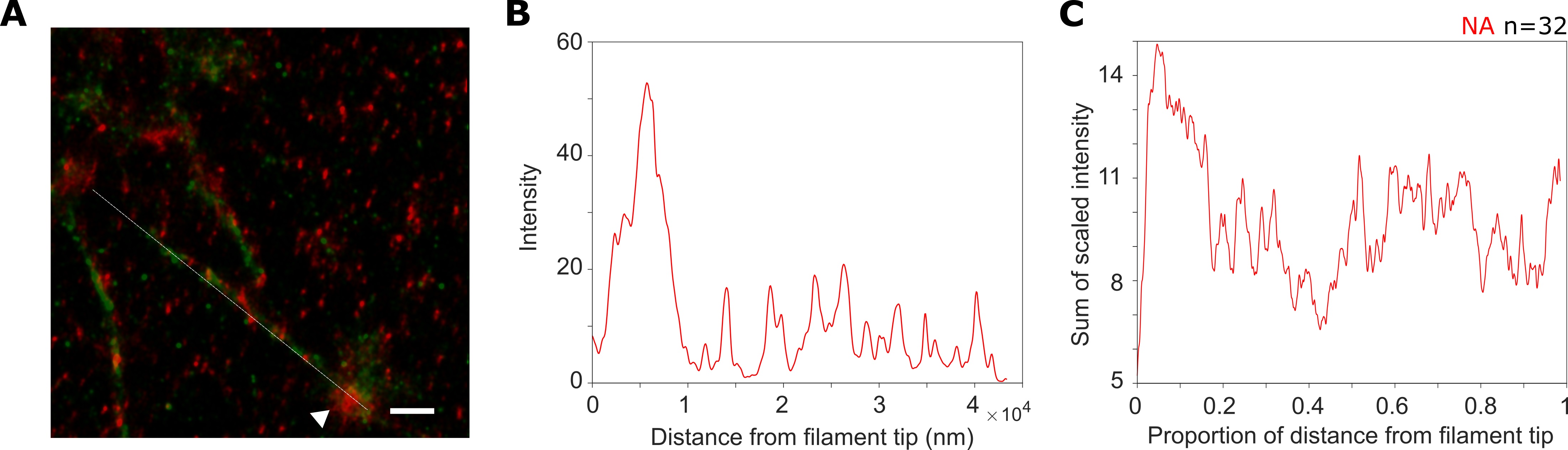

Supplement: S9 Fig — A) Diffraction limited image of A/Udorn/72 filaments stained with for HA (green) and NA (red) budding out of an infected cell. White line denotes a filament with an Archetti body (arrow) at the tip. Scale bar 1 μm. B) Intensity trace of the NA signal from the filament highlighted in A), showing higher NA signal at the Archetti body at the tip. C) Normalised averaged intensity traces of the NA signal from filaments without obvious Archetti body structures at the tip, showing an enrichment at the filament ends furthest from the cell membrane. (JPG) [file ppat.1011484.s009.jpg]

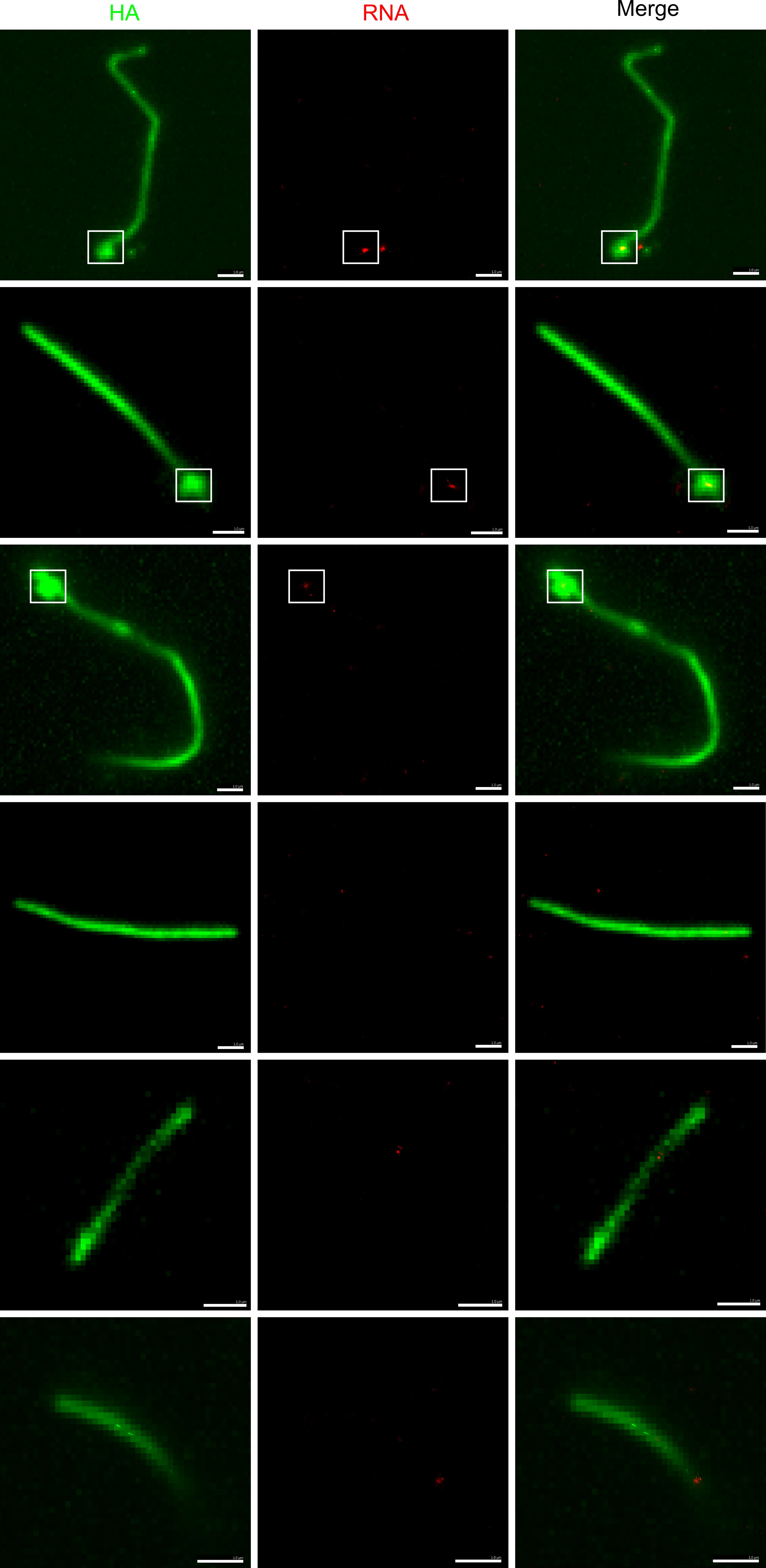

Supplement: S10 Fig — Diffraction limited images of filamentous A/Udorn/72 stained with an anti-HA antibody (green; left panels), super-resolution localisations from an array of FISH probes against the NA gene segment (red; middle panels), and merged images (right panels). White boxes denote Archetti bodies. Scale bars 1 μm. (JPG) [file ppat.1011484.s010.jpg]

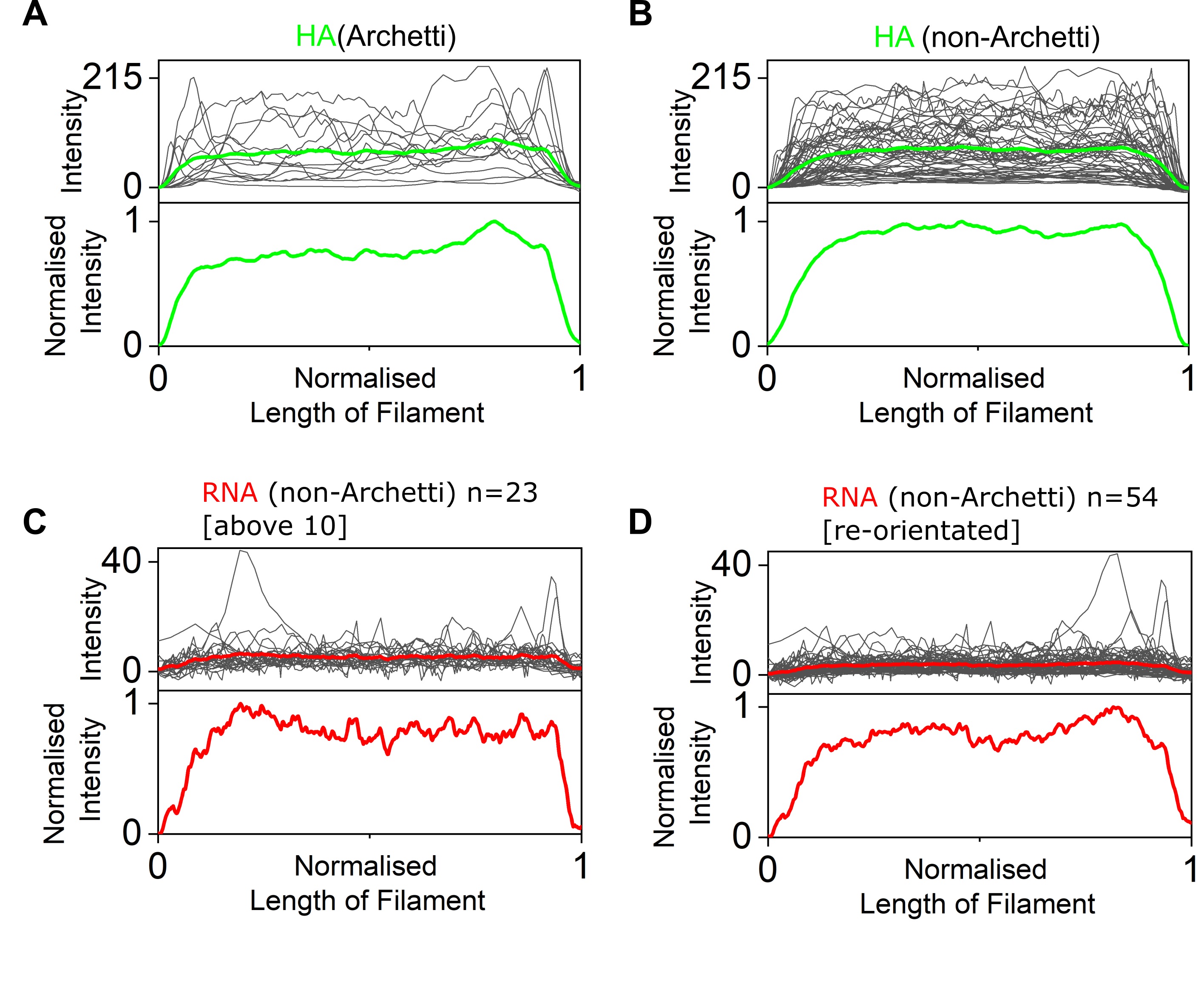

Supplement: S11 Fig — A) Top: Raw intensity traces (grey) of the HA signal from 14 filaments with a visible Archetti body, with the average intensity profile shown as a green line. Bottom: Normalised average RNA signal from the 14 filaments, showing peak in intensity of the HA signal in Archetti bodies. B) Same as A) but for the HA signal from 54 filaments with no visible Archetti body. C) Top: Raw intensity traces (grey) of the RNA signal from 23 filaments with intensity peaks above an arbitrary background threshold of 10, with the average intensity profile shown as a red line. Bottom: Normalised average RNA intensity from the 23 filaments. D) Same as C) but for the RNA signal from all non-Archetti filaments that have been re-orientated to line up the largest peaks on one side. (JPG) [file ppat.1011484.s011.jpg]
